# Core and surface structure and magnetic properties of mechano-synthesized LaFeO_3_ nanoparticles and their Eu^3+^-doped and Eu^3+^/Cr^3+^-co-doped variants

**DOI:** 10.1038/s41598-024-65757-z

**Published:** 2024-06-26

**Authors:** R. T. Al-Mamari, H. M. Widatallah, M. E. Elzain, A. M. Gismelseed, A. D. Al-Rawas, S. H. Al-Harthi, M. T. Z. Myint, N. Al-Saqri, M. Al-Abri

**Affiliations:** 1 Physics Department, Sultan Qaboos University, Al-Khoudh, P.O. Box 36, Muscat, 123 Oman; 2 Nanotechnology Research Center, Sultan Qaboos University, Al-Khoudh, P.O. Box 17, Muscat, 123 Oman

**Keywords:** Orthoferrites, Mechano-synthesis, XRD, Mössbauer spectroscopy, XPS, Van-Vleck paramagnetism, Materials science, Nanoscience and technology, Physics

## Abstract

The core and surface structure and magnetic properties of mechano synthesized LaFeO_3_ nanoparticles (30–40 nm), their Eu^3+^-doped (La_0.70_Eu_0.30_FeO_3_), and Eu^3+^/Cr^3+^ co-doped (La_0.70_Eu_0.30_Fe_0.95_Cr_0.05_O_3_) variants are reported. Doping results in a transition from the *O*′*-type* to the *O-type* distorted structure. Traces of reactants, intermediate phases, and a small amount of Eu^2+^ ions were detected on the surfaces of the nanoparticles. The nanoparticles consist of antiferromagnetic cores flanked by ferromagnetic shells. The Eu^3+^ dopant ions enhance the magnetization values relative to those of the pristine nanoparticles and result in magnetic susceptibilities compatible with the presence of Eu^3+^ van Vleck paramagnetism of spin–orbit coupling constant (λ = 363 cm^−1^) and a low temperature Curie–Weiss like behavior associated with the minority Eu^2+^ ions. Anomalous temperature-dependent magnetic hardening due to competing magnetic anisotropy and magnetoelectric coupling effects together with a temperature-dependent dopant-sensitive exchange bias, caused by thermally activated spin reversals at the core of the nanoparticles, were observed.

## Introduction

The orthorhombically distorted perovskite-related rare-earth orthoferrite materials of the composition (RE)FeO_3_, where RE is a rare earth ion, are technologically attractive due to their physicochemical properties that render them of potential usage in a host of applications^1–7^. One such a material is lanthanum orthoferrite, LaFeO_3_, that crystallizes in the space group *Pbnm* (# 62) with lattice constants *a* = 5.556 Å, *b* = 5.565 Å and *c* = 7.862 Å^5^. The La^3+^ and Fe^3+^ ions, respectively, occupy the so-called A sites and the six O^2−^ coordinated octahedral B sites (BO_6_). Unlike cubic perovskites, where the A sites are 12 O^2−^-coordinated cuboctahedra (AO_12_), the orthorhombically distorted (RE)FeO_3_ (RE = from Pr to Lu) compounds possess 8 O^2−^-coordinated A sites (AO_8_). However, in *bulk* LaFeO_3_, Marezio et al.^8^ have shown that the difference between the distances of the La^3+^ ion to its eighth and ninth nearest O^2−^ neighbors, namely, 2.805 Å and 3.041 Å, respectively, is insignificant. Hence, the A sites in LaFeO_3_ are considered AO_9_ polyhedra. The magnetic ordering of the high-spin Fe^3+^ cations in LaFeO_3_ results in G-*type* antiferromagnetism (AFM) which is accompanied by a weak canted ferromagnetic (FM) component. The high Néel temperature (T_N_) of ~ 740 K reported for the material is a consequence of the strong Fe^3+^–O^2−^–Fe^3+^ superexchange coupling^1,9^. The other inherent properties of LaFeO_3_, which are important from an applied viewpoint, include thermal, electrical, magneto-optical, and high chemical stability, in addition to multiferroicity^2,4,5,10^.

To extend the technological utilization of LaFeO_3_, some researchers have attempted to modify its properties by producing it either in the form of nanoparticles or by introducing substituents for La^3+^ and/or Fe^3+^ cations^2–4^. In this respect, novel magnetic properties, such as magnetic exchange bias (*EB*), superparamagnetism, and spin glass behavior, have been reported for LaFeO_3_ nanoparticles of sizes in the 10–60 nm range^2,3,9–11^. Such properties, which are of interest for various applications, including magnetic sensors, spintronics, and data storage devices^1,4,6^, could be further tuned by single-site cation doping. In connection with this, we note that when soft chemistry methods were used to introduce Na^+^, Zn^2+^, Sb^3+^ or Ce^4+^ as sole substituents for La^3+^ in LaFeO_3_ nanoparticles, the coercivity (*H*_*c*_) was found to depend on the dopant concentration^3,5,11^. Doping with single cations such as Na^+^ and Zn^2+^ has affected the superparamagnetic behavior of the nanoparticles, as both the blocking and relaxation temperatures were found to decrease^3,9^. Of interest to us in this paper is the work of Hosseini et al. who reported the use of the sol–gel route to form Eu^3+^-doped LaFeO_3_ nanoparticles of the composition La_1*−x*_Eu_*x*_FeO_3_ (*x* ≤ 0.15) without elaborating on how Eu^3+^-doping affects their structural and magnetic properties^12^. Similarly, the substitution of Fe^3+^ in LaFeO_3_ nanoparticles with transition metal (TM) cations has been an active area of research^4,6,10^. For instance, a spin-glass-like freezing temperature and cluster-spin behavior were reported when Mn^3+^ and Cr^3+^ were used as substituents for Fe^3+^ in LaFeO_3_ nanoparticles^13,14^. *EB* behavior has been reported to be barely noticeable when substituting La^3+^ cations with Zn^2+^ in LaFeO_3_ nanoparticles^3,10^. Ferromagnetically weak single-domain LaFeO_3_ nanoparticles with particle size-dependent *H*_*c*_ were reported when Fe^3+^ ions were partially substituted^4^ by Ti^4+^. Recently, we have shown that Ru^3+^-doping modifies the properties of mechano-synthesized LaFe_1−*x*_Ru_*x*_O_3_ nanoparticles, resulting in Jahn–Teller-like distortion, a size-dependent hyperfine magnetic field, and a monotonic decrease in the optical band gap with increasing Ru^3+^ content^6^. In addition, the structure of the mechano-synthesized LaFe_1−*x*_Ru_*x*_O_3_ nanoparticles was found to index to the* O*′*-type* perovskite structure with the lattice parameters related according to c/$\sqrt 2$ < *a* < *b* as opposed to the *O-type* perovskite structure of bulk LaFeO_3_ wherein *a* < c/$\sqrt 2$ < *b*^6^. These modifications are associated with the route of mechano-synthesis, which is known to form nanoparticles with a non-uniform core–shell structure in which the exchange interactions between the inner crystalline core and an outer disordered or amorphous shell often result in novel physical properties^15,16^. To the best of our knowledge, few studies have been devoted to the case in which both La^3+^ and Fe^3+^ cations in LaFeO_3_ nanoparticles are concurrently substituted^17–19^. Such co-doping was found to lead to notable changes in the magnetic and ferroelectric properties, as was shown for the La_0.8_Sr_0.2_Fe_1−*x*_Cu_*x*_O_3_, La_0.9_Dy_0.1_Fe_0.9_Ti_0.1_O_3_ and La_1−*x*_Dy_*x*_Fe_1−*y*_Mn_*y*_O_3_ nanoparticles. In a previous study on mechano-synthesized nanoparticles of the isostructural compound EuFeO_3_, co-doped with Nd^3+^ and Cr^3+^ with the composition Nd_0.33_Eu_0.67_Fe_1−*x*_Cr_*x*_O_3_, we reported an unusual crystal distortion wherein Eu^3+^ and Fe^3+^ cations exchange their normal expected A and B sites and novel magnetic properties^15^.

In this paper, we report on the synthesis of LaFeO_3_, La_0.7_Eu_0.3_FeO_3_ and La_0.7_Eu_0.3_Fe_0.95_Cr_0.05_O_3_ nanoparticles using a mechanical milling route. We then systematically study the effect of Eu^3+^-doping and Eu^3+^/Cr^3+^ co-doping on the structural and magnetic properties of the synthesized nanoparticles. Of special interest to us is studying the contribution of the majority Eu^3+^ and minority Eu^2+^ ions detected on the intrinsic magnetic properties of the nanoparticles. As the investigated compounds have AFM ground states, it is important to carefully synthesize them as single phases so as to eliminate any contribution of Fe or Fe-oxide impurities on these magnetic properties. The experimental techniques used include X-ray diffraction (XRD), Fourier transform infrared spectroscopy (FT-IR) and ^57^Fe Mӧssbauer spectroscopy, X-ray photoelectron spectroscopy (XPS) and vibrating sample (VSM) magnetometery.

## Materials and methods

LaFeO_3_, La_0.70_Eu_0.30_FeO_3_ and La_0.70_Eu_0.30_Fe_0.95_Cr_0.05_O_3_ nanoparticles were prepared starting from stoichiometric mixtures of high-purity α-Fe_2_O_3_, La_2_O_3_, Eu_2_O_3_ and Cr_2_O_3_ that were subjected to mechanical milling for different times using a Fritch D-55743 P6 milling machine with tungsten carbide vial (250 mL) and balls. The milling speed was 300 rpm, and the ball-to-powder mass ratio was 15: 1. A Carbollite (HTF 1800) furnace was used to heat the pre-milled mixtures for 10 h at various temperatures to achieve single-phased final products. XRD measurements were performed using an X’Pert PRO PANanalytical diffractometer where the Cu-K_α_ radiation (λ = 1.5406 Å) was employed in the 2θ-range of 20.00–80.00° at the rate of 0.02° per second. The GSAS program was used to perform XRD Rietveld refinements^20^. A JEM-1400-JEOL system, operating at 200 kV, was used to obtain high-resolution transmission electron microscopy (HRTEM) images. A PerkinElmer SpectraOne system was used to collect FT-IR spectra in the range 500–4000 cm^−1^ with a signal resolution of 4 cm^−1^ for 40 scans. ^57^Fe Mössbauer measurements were done at 298 K and 78 K with a conventional constant acceleration spectrometer using the 14.4 keV gamma ray provided by a 50 mCi ^57^Co/Rh source operating in transmission mode. The isomer shift values are quoted relative to α-Fe at 298 K. An Omicron NanoTechnology MXPS system (Scienta Omicron, Germany) employing the Al Kα radiation (*hν* = 1486.6 eV) was used to collect XPS spectra. The C 1*s* reference peak, at the binding energy of 284.6 eV, was used for binding energy calibration, and the data was fitted with the CasaXPS^21^. The VSM magnetometer option of a Quantum Design PPMS system was used to record the thermal dependence of magnetization. The measurements were carried out in both the field cooling (FC) and zero field cooling (ZFC) modes under an external magnetic field of 50 kOe in the 4–300 K temperature range. ZFC magnetic hysteresis loops were obtained at different temperatures and applied fields of up to 9 T.

## Results and discussion

### Mechano-synthesis and crystal structure

**Figure 1:**
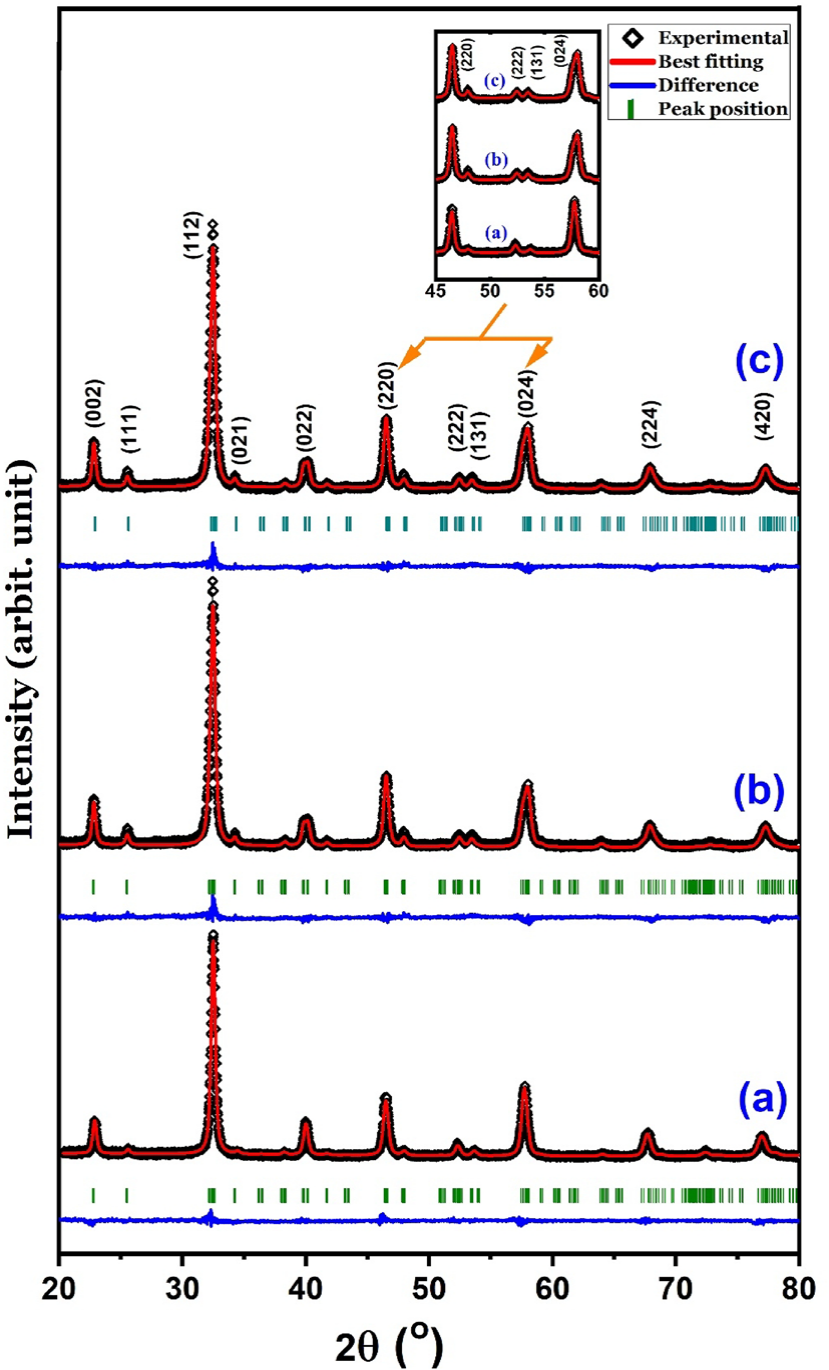
Observed, calculated and difference of the XRD patterns for the (**a**) LaFeO_3_, (**b**) La_0.70_Eu_0.30_FeO_3_, and (**c**) La_0.70_Eu_0.30_Fe_0.95_Cr_0.05_O_3_ samples. The Miller indices of each peak are given in the form of (*hkl*) and the blue bars indicate the positions of Bragg’s reflection peaks. The inset shows the relative intensity reversal of the (220) and (024) peaks.

**Figure 2:**
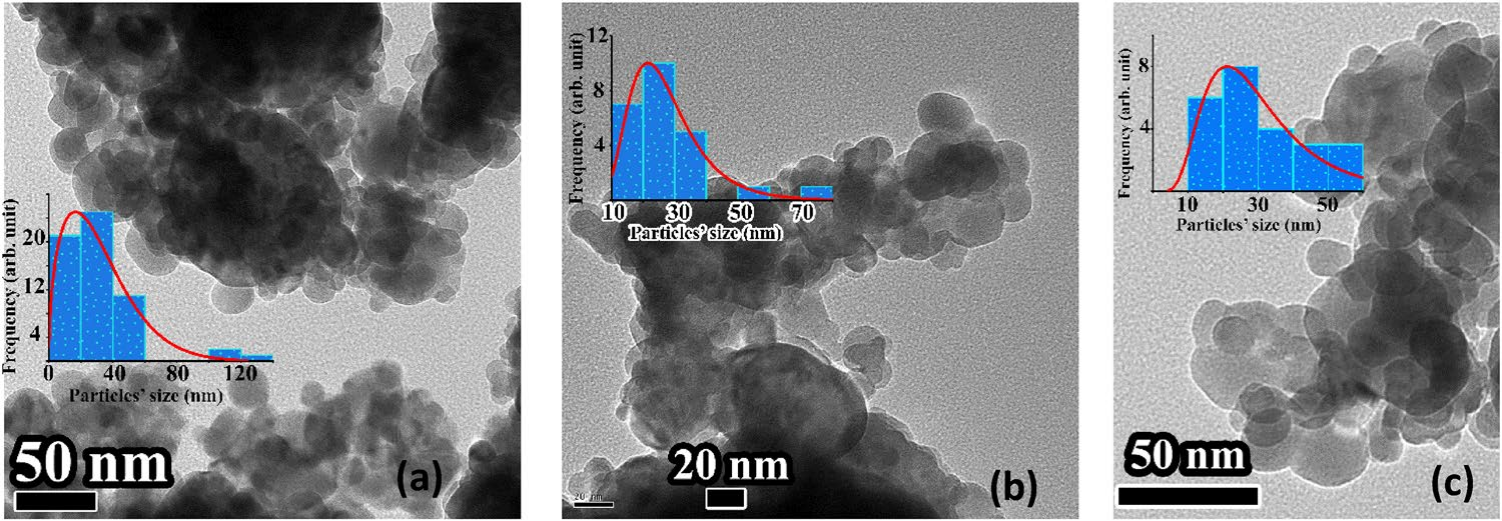
TEM images of the (**a**) LaFeO_3_, (**b**) La_0.70_Eu_0.30_FeO_3_, and (**c**) La_0.70_Eu_0.30_Fe_0.95_Cr_0.05_O_3_ nanoparticles. The insets are the histograms of the particle size distribution. The solid line is the fit to the log-normal size distribution.

**Figure 3:**
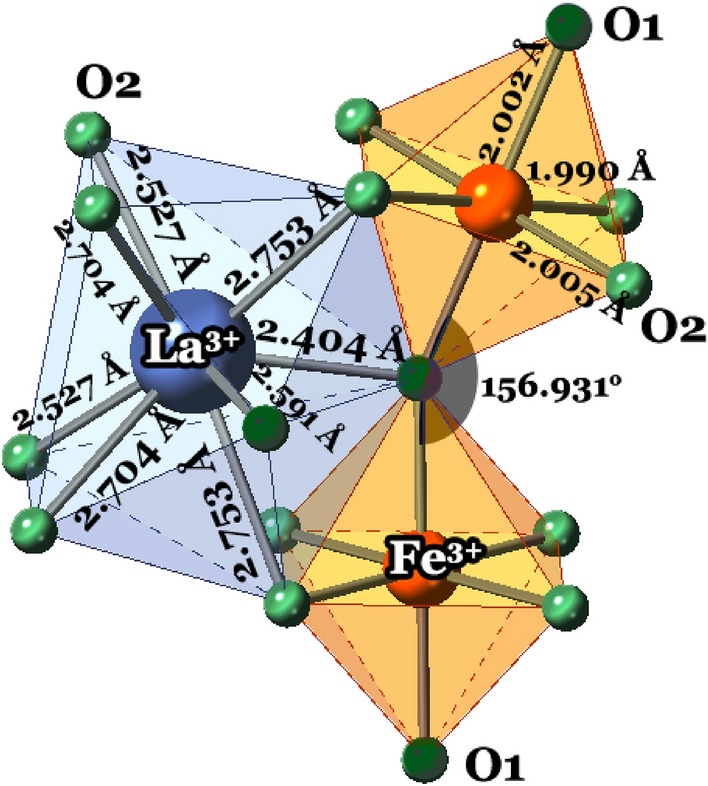
The polyhedra of the LaFeO_3_ nanoparticles as derived from the XRD Rietveld refinement. The large gray spheres represent La^3+^ ions in 8 O^2−^ coordinated sites, the orange spheres refer to Fe^3+^ ions in FeO_6_ octahedra and the small green spheres represent O^2−^ ions. O1 and O2 refer to O^2−^ ions with two different environments.

The XRD patterns shown in Fig. 1 indicate that the formation of single-phase LaFeO_3_ and that of its Eu^3+^-doped LaFeO_3_ and Eu^3+^/Cr^3+^ co-doped LaFeO_3_ (La_0.70_Eu_0.30_Fe_0.95_Cr_0.05_O_3_) variants are attained after heating the corresponding 80 h pre-milled reactants’ mixtures at 600 °C (10 h) and 700 °C (10 h), respectively. These temperatures are *ca.* 600–700 °C lower than the ones reported for the formation of LaFeO_3_ and its cation-doped modifications using the conventional solid-state routes^22^. It is obvious from the TEM images shown in Fig. 2 that the three materials are composed of semi-spherical nanoparticles that tend to agglomerate. The LaFeO_3_ sample exhibits the widest particle size distribution with the mean particle size of (40 ± 10) nm relative to both La_0.70_Eu_0.30_FeO_3_ and La_0.70_Eu_0.30_Fe_0.95_Cr_0.05_O_3_ (30 ± 10) nm. The results of the XRD Rietveld refinements, where in the case of the doped samples a common position for the La^3+^ and Eu^3+^ ions was assumed and a temperature factor to account for their disorder was included, are also shown in Fig. 1, Tables 1 and 2. It follows that each nanomaterial is structurally indexable to an orthorhombic perovskite-related phase (space group *Pbnm*)^5,7^. As the microstrain values for the doped LaFeO_3_ samples (Table 2) are too small to be considered a factor contributing to the structural distortion associated with apparent broadened peaks relative the ones expected for the corresponding bulk samples, we attribute the broadening to lattice dislocations and surface disorder associated with crystallite size reduction to the nanometer scales^23^. The relatively large values of the microstrain in the La_0.70_Eu_0.30_FeO_3_ and La_0.70_Eu_0.30_Fe_0.95_Cr_0.05_O_3_ samples, relative to that of the LaFeO_3_ sample, are obviously induced by cationic doping and co-doping. The data given in Tables 1 and 2 were used in combination with VESTA software^24^ to draw the polyhedra of the crystal structure of LaFeO_3_ with the ionic positions, bond lengths, and angles shown in Fig. 3.

**Table 1:** The structural parameters extracted from the Rietveld refinement of XRD data for the LaFeO_3_, La_0.70_Eu_0.30_FeO_3_ and La_0.70_Eu_0.30_Fe_0.95_Cr_0.05_O_3_ nanoparticles recorded at room temperature. Space group: *Pbnm* (62) and the R-Factors have their normal significance and relate to regions of the profile at which Bragg peaks contribute. R_p_ < 7.91%, R_wp_ < 7.99%, R_exp_ < 6.77%, *χ*^*2*^ ~ *1.483.*

Parameter	LaFeO_3_	La_0.70_Eu_0.30_FeO_3_	La_0.70_Eu_0.30_Fe_0.95_Cr_0.05_O_3_
*a *(Å)	5.5549(2)	5.5019(5)	5.5006(2)
*b* (Å)	5.5608(1)	5.5682(2)	5.5671(4)
*c* (Å)	7.8472(3)	7.8089(6)	7.8067(1)
c/2 (Å)	5.5488(2)	5.5217(5)	5.5202(3)
Volume (Å)^3^	242.396(1)	239.234(3)	239.056(4)
Calc. density (g/cm^3^)	6.652(4)	6.849(1)	6.848(5)
Crystallite size (nm)	31(1)	28(1)	36(1)
Microstrain (%)	0.000(2)	0.20(2)	0.58(3)
(Fe/Cr)-O1 (Å)	2.002(9)	1.9818(2)	1.9705(2)
(Fe/Cr)-O2 (Å)	1.990(3)	1.9120(2)	1.9243(2)
(Fe/Cr)-O2 (Å)	2.005(3)	2.1060(2)	2.1209(2)
(Fe/Cr)-O1-(Fe/Cr) (°)	156.931(1)	160.182(2)	164.130(2)
(Fe/Cr)-O2-(Fe/Cr) (°)	161.621(1)	153.827(2)	150.595(3)
O1-(Fe/Cr)-O2 (°)	89.4, 87.7	85.3, 89.4	79.4, 84.9
O2-(Fe/Cr)-O2 (°)	89.2, 90.8	88.2, 91.8	87.1, 92.9
(Fe/Cr)O_6_ volume (Å)^3^	10.569 (2)	10.599(4)	10.493(3)

(Fe/Cr)-O is bond length; (Fe/Cr)-O-(Fe/Cr) and O-(Fe/Cr)-O are angles. The O2 oxygen ions are equatorial to Fe^3+^, while the O1 oxygen ions are axial.

**Table 2:** The fractional coordinates of the ions in the LaFeO_3_, La_0.70_Eu_0.30_FeO_3_ and La_0.70_Eu_0.30_Fe_0.95_Cr_0.05_O_3_ nanoparticles extracted from the Rietveld refinement of the XRD data obtained at room temperature.

Ion and site/structural parameter	LaFeO_3_	La_0.70_Eu_0.30_FeO_3_	La_0.70_Eu_0.30_Fe_0.95_Cr_0.05_O_3_
La^3+^ in 4c(x y ¼)			
x/a	− 0.0029(1)	− 0.0098(1)	− 0.0093(2)
y/b	0.0250(4)	0.0359(4)	0.0366(2)
Eu^3+^ in 4c(x y ¼)			
x/a	–	− 0.0098(1)	− 0.0093(2)
y/b	–	0.0359(4)	0.0366(2)
Occupancy of La^3+^:Eu^3+^	1.00:0.00		
Fe^3+^/Cr^3+^ in 4b(0 ½ 0)			
Occupancy of Fe^3+^:Cr^3+^	1.00:0.00		
O^2−^ (1) in 4c(x y ¼)			
x/a	0.0591(1)	0.0574(1)	0.0411(1)
y/b	0.4764(4)	0.4768(3)	0.4763(5)
O^2−^(2) in 8d(x y z)			
x/a	− 0.2731(3)	− 0.2772(1)	− 0.2665(3)
y/b	0.2852(1)	0.3017(6)	0.2960(4)
z/c	0.0364(4)	0.0426(5)	0.0590(5)

For the LaFeO_3_ nanoparticles, the difference between the distances of La^3+^ to its eighth and ninth nearest O^2−^ neighbors, 2.527 Å and 3.028 Å, is larger than that expected for bulk LaFeO_3_ mentioned earlier^8,10^. This, in turn, justifies the conclusion that the La^3+^ polyhedron at the A-site of the LaFeO_3_ nanoparticles is LaO_8_ rather than LaO_12_ as expected for *bulk* LaFeO_3_^8,10^. This result indicates that the change in the La^3+^ coordination number is strongly instigated by the weakening of ionic exchange interactions at the surface of the nanoparticles. From Table 1, it can be seen that the Fe–O1 bond lengths vary coincidentally with the FeO_6_-volumes. There was no cationic site exchange similar to that we previously reported for EuFe_*x*_Cr_1−*x*_O_3_ nanocrystalline particles using a similar mechano-synthesis regime^25^. The fact that the lattice constants of the LaFeO_3_ nanoparticles are slightly smaller than those of their bulk counterparts cited above^22^ may be attributed to the dislocations and disorder induced on the surface layers of the nanoparticles. The FeO_6_ octahedra in the LaFeO_3_ nanoparticles (Fig. 3) appeared to be severely distorted owing to variations in the *intra*-octahedral O1–Fe–O2 bond angles.

It is clear from the obtained lattice parameters of the LaFeO_3_ nanoparticles that their distorted crystal structure is of the *O*′*-type*^6^. The diffraction peaks of the La_0.70_Eu_0.30_FeO_3_ nanoparticles broadened and shifted toward larger angles (Fig. 1). Hence, their lattice constants decreased relative to those of the LaFeO_3_ nanoparticles (Table 1). These changes entail the substitution of 8-coordinated La^3+^ ions (1.36 Å) with smaller 8-coordinated Eu^3+^ ions (1.066 Å) in the lattice structure of LaFeO_3_^26^. Generally, the influence of the Eu^3+^ dopant on the deformation of octahedral sites is justifiable in terms of the Goldsmith formalism^7^. In contrast to the LaFeO_3_ nanoparticles, it can be seen from Table 1 that doping with Eu^3+^ affects the lattice constants of the La_0.70_Eu_0.30_FeO_3_ nanoparticles, such that *a* < c/$\sqrt 2$ < *b* indicates that the orthorhombic distortion of the nanoparticles is *O-type*. We associate this crossover of the* a* and c/$\sqrt 2$ parameters with the significant difference between the O1-Fe-O2 angle in the Eu^3+^-doped LaFeO_3_ nanoparticles and LaFeO_3_ nanoparticles (Table 1) owing to the partial substitution of La^3+^ ions with smaller Eu^3+^ ions. Similar anomalous parameter crossovers have been reported for solid solutions of rare-earth orthoferrites and orthocobaltites, such as PrCo_1−*x*_Fe_*x*_O_3_ and EuCo_1−*x*_Fe_*x*_O_3_^27,28^. To reflect more on the structural distortion resulting from Eu^3+^ doping, using Glazer's tilt system of perovskites^29^, one notes that the variation in the lattice parameters of the La_0.70_Eu_0.30_FeO_3_ nanoparticles corresponds to the first tilt type along the [010] direction. As shown in Table 1, partial substitution of La^3+^ with Eu^3+^ led to a single long Fe-O2 bond along the *b*-axis and a pair of Fe-O1 and Fe-O2 short bonds in the *ac* plane.

We now turn to La_0.70_Eu_0.30_Fe_0.95_Cr_0.05_O_3_ nanoparticles. As shown in Table 2, the Eu^3+^ and La^3+^ ions preferentially occupy a slightly distorted “average” A-site, whereas the Cr^3+^ ions are randomly distributed with Fe^3+^ ions in the B-sub-lattice.. It turns out that the introduction of 5% Cr^3+^ (0.615 Å) substituent ions for Fe^3+^ (0.645 Å)^26^ resulted in a slight increase in the *a* lattice parameter and a concomitant decrease in the *b* and* c* lattice parameters relative to those of the La_0.70_Eu_0.30_FeO_3_ nanoparticles. The estimated crystallite sizes for the three types of nanoparticles were within experimental error, consistent with the particle sizes deduced from the TEM measurements. This implies that the nanoparticles may generally be considered crystallites.

**Figure 4:**
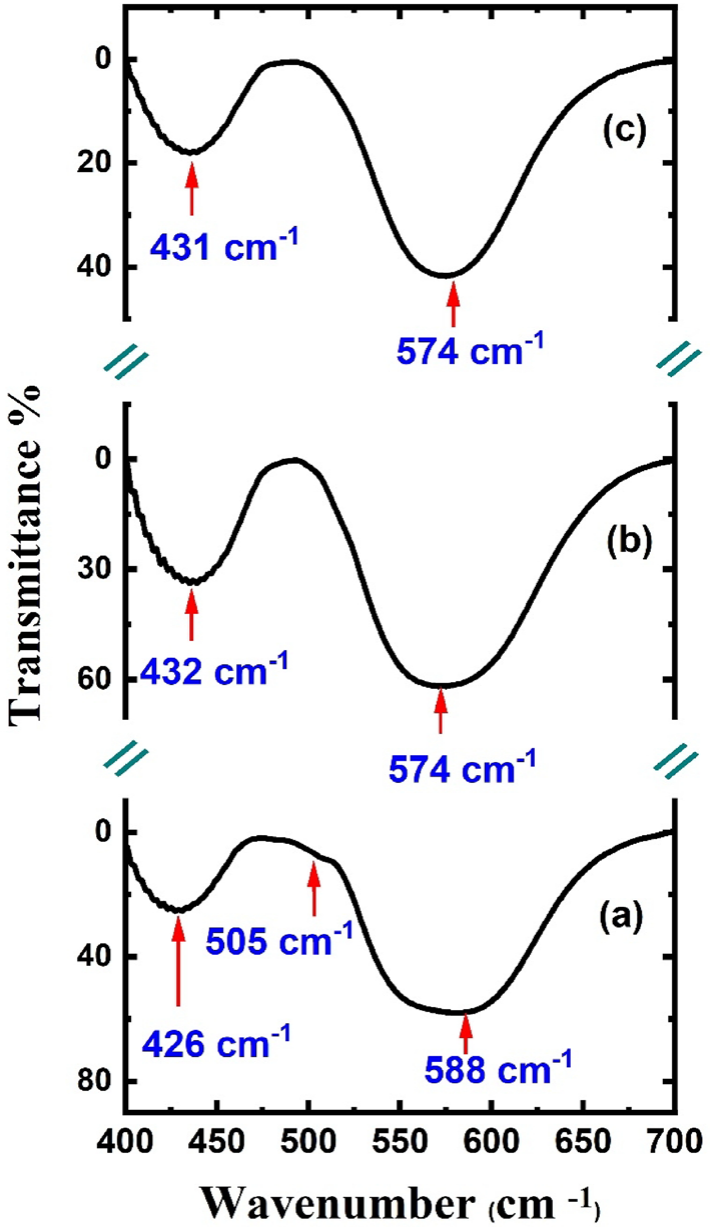
FT-IR spectra of the (**a**) LaFeO_3_, (**b**) La_0.70_Eu_0.30_FeO_3_, and (**c**) La_0.70_Eu_0.30_Fe_0.95_Cr_0.05_O_3_ nanoparticles in the range of 400–700 cm^−1^ at 298 K.

The FT-IR spectra of the mechano-synthesized LaFeO_3_ nanoparticles and their Eu^3+^-doped and Eu^3+^/Cr^3+^-co-doped modifications in the range of 400–700 cm^−1^ shown in Fig. 4 are typical of perovskite oxides that structurally index to the *Pbnm* space group^4,7^. Theoretically, *Pbnm* phases have nine dipole-active optical phonon modes in the 400–700 cm^−1^ range, of which those between 400 and 500 cm^−1^ are O^2−^ octahedral bending vibrations, and those beyond 500 cm^−1^ are O^2−^ stretching vibrations^7,30^. However, some peaks are very difficult to detect because of the line broadening associated with the small particle sizes or the possible presence of O^2−^ vacancies on the surfaces. Evidently, the slight differences in the FT-IR spectra relative to the spectrum of the LaFeO_3_ nanoparticles were a consequence of cationic doping. On the basis of the ionic interactions present, the vibrational bands would contribute intrinsic Lorentzian shapes to the FT-IR spectrum. Temperature-dependent effects, instrumental effects, and/or sample characteristics cause the background signals to experience Gaussian broadening^31,32^. Hence, we opted to fit the FT-IR spectra shown in Fig. 4 with the Voigt functions. The spectral broadband at ~ 588 cm^−1^, for the LaFeO_3_ nanoparticles was assigned to the antisymmetric stretching vibrational modes of the FeO_6_ octahedra^7^. The relatively asymmetric peak may be related to the non-uniform cationic distribution in the shells of the nanoparticles, which leads to cluster-glass-like features^14^. Peak broadening reflects the wide size distribution observed in the TEM image (Fig. 2). The weak band at *ca.* 505 cm^−1^ owing to the out-of-phase stretching vibrations of the BO_6_ octahedra, as has been observed for some orthochromites and orthomanganites^32^, is closely associated with a Jahn–Teller-like distortion^30^. For Eu^3+^-and Eu^3+^/Cr^3+^-doped LaFeO_3_ nanoparticles, the notable enhancement in the symmetry of the stretching vibrational modes and their narrowing are indicative of a more uniform cationic distribution and limited particle size range relative to those of the LaFeO_3_ nanoparticles. The substitution of the lighter La^3+^ ions (138.904 amu) by heavier Eu^3+^ ions (151.962 *amu*) in the La_0.70_Eu_0.30_FeO_3_ nanoparticles shifted the absorption band of the FeO_6_ stretching vibrations from 583 to 574 cm^−1^, which is expected, as the wavenumber is inversely proportional to the ionic mass^18,33^. The small concentration of Cr^3+^ ions in the La_0.70_Eu_0.30_Fe_0.95_Cr_0.05_O_3_ nanoparticles was not sufficient to cause an observable shift for the same band relative to that of the La_0.70_Eu_0.30_FeO_3_ nanoparticles. The small shoulder at 505 cm^−1^, associated with the *O*′*-type* structural mode of LaFeO_3_ does not exist in the Eu^3+^-doped and Eu^3+^/Cr^3+^-co-doped nanoparticles, presumably because of the *O-type* structural distortion. We now turn to The shifts in the bending vibrational mode at 426 cm^−1^ in the FT-IR spectrum of the LaFeO_3_ nanoparticles to 432 cm^−1^ and 431 cm^−1^ in the spectra of La_0.70_Eu_0.30_FeO_3_ and La_0.70_Eu_0.30_Fe_0.95_Cr_0.05_O_3_, respectively. These shifts are consistent with the increased bending of the Fe–O2–Fe angle revealed by the XRD refinement of the La_0.70_Eu_0.30_FeO_3_ nanoparticles and the subsequent decrease in the electronegativity of Cr^3+^–O^2−^ compared with Fe^3+^–O^2−^ in the La_0.70_Eu_0.30_Fe_0.95_Cr_0.05_O_3_ nanoparticles. Hence, the FT-IR spectra further confirmed that LaFeO_3_ nanoparticles and their Eu^3+^-doped and Eu^3+^/Cr^3+^ co-doped modifications were formed.

### Mössbauer characterization

**Figure 5:**
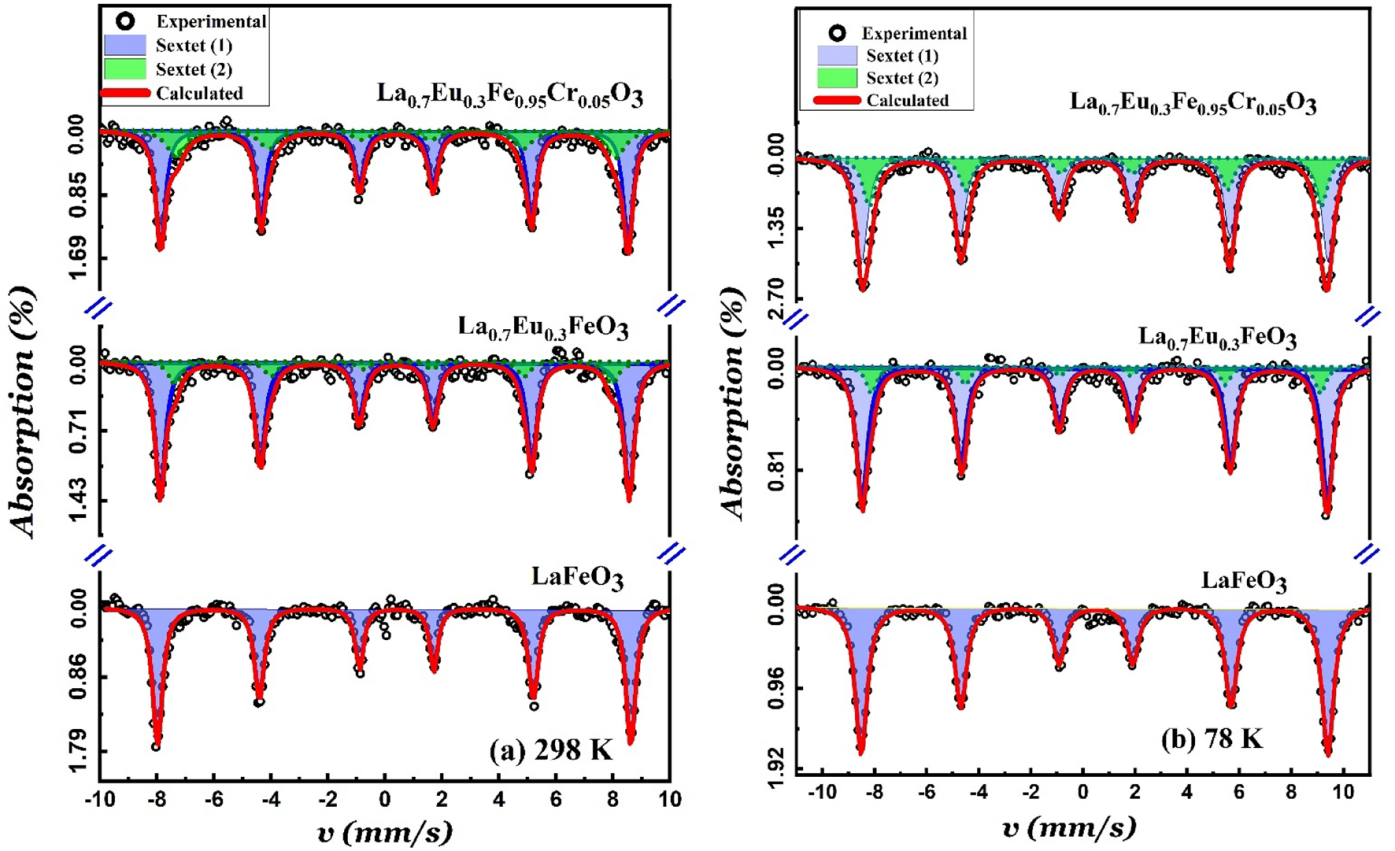
The ^57^Fe Mӧssbauer spectra recorded from the (**a**) LaFeO_3_, (**b**) La_0.70_Eu_0.30_FeO_3_, and (**c**) La_0.70_Eu_0.30_Fe_0.95_Cr_0.05_O_3_ nanoparticles at (**a**) 298 K and (**b**) 78 K.

The zero-field ^57^Fe Mössbauer spectra of LaFeO_3_, La_0.70_Eu_0.30_FeO_3_ and La_0.70_Eu_0.30_Fe_0.95_Cr_0.05_O_3_ nanoparticles, recorded at 298 K and 78 K, are shown in Fig. 5, and the corresponding fitted hyperfine parameters are given in Table 3. All spectra show pure magnetic six-line patterns with broadened absorption lines, which reflect both the particle size distribution and varied cationic environments for the doped samples around the ^57^Fe nuclei. It is pertinent to note that Fujii et al.^1^ reported Mössbauer spectra for LaFeO_3_ nanoparticles with an average particle size of *ca.* 13 nm, which are pure doublets. While the LaFeO_3_ system is essentially AFM in character, the Mössbauer doublets were attributed to the superparamagnetic behavior of the FM nanoparticle shells that resulted from the uncompensated surface spins^1^. The absence of such doublets in the Mössbauer spectra of the present nanoparticles rules out the presence of any superparamagnetic behavior, which is consistent with their relatively larger average particle sizes relative to 13 nm^1,14^. The Mössbauer spectrum of the LaFeO_3_ nanoparticles at either 298 or 78 K was best fitted to a single sextet with a typical isomer shift value of the Fe^3+^ state^11,34^. The isomer shift values at 298 K (0.37–0.41 mm/s) indicate the high-spin nature of the Fe^3+^ ions in all nanoparticles^6^. The increase in the isomer shift values at 78 K relative to their values at 298 K is explicable in terms of the second-order Doppler shift^34^. The small negative quadrupole shift values at both temperatures are indicative of the distorted crystal structures, as discussed earlier^6,11^. We associate the relatively high value of the hyperfine magnetic field of the LaFeO_3_ sample at 298 K (51.5 T), which is typical for nanoparticles exhibiting AFM ordering with strong Fe–Fe coupling, with Fe nuclei at the nanoparticle cores^1,6^. The tangible increase in the hyperfine field value as the temperature is decreased to 78 K (Table 3) is associated with the removal of the thermal vibrations and subsequent spin alignment, especially in the shells of the nanoparticles^1,13,15,34^. As shown in Fig. 5 and Table 3, doping the LaFeO_3_ nanoparticles with Eu^3+^ and co-doping them with Eu^3+^/Cr^3+^ broadened the Mössbauer absorption lines. The best fit of the spectra of both doped nanoparticles was attained using two superimposed magnetic subspectra, S1 and S2. S1, with smaller isomer shifts and larger effective hyperfine magnetic fields, are associated with Fe^3+^ environments similar to those in undoped LaFeO_3_ or with poor Eu^3+^ (in La_0.70_Eu_0.30_FeO_3_) and Eu^3+^/Cr^3+^ (in La_0.70_Eu_0.30_Fe_0.95_Cr_0.05_O_3_) environments. The slight decrease in the hyperfine field values of sextet S1 at both temperatures for both types of doped nanoparticles relative to those of the LaFeO_3_ nanoparticles is attributed to their relatively smaller average particle size, as revealed by the TEM images. Slight differences existed between the isomer shift values of the undoped LaFeO_3_ nanoparticles and those of the S1 sextet. This may be related to the different electronic configurations of the dopant ions that, in turn, lead to slightly different electric fields at the sites of ^57^Fe nuclei^34^. Sextet S2, whose hyperfine magnetic fields are notably smaller than those of the LaFeO_3_ nanoparticles, is associated with Fe^3+^ environments where the Fe–O–Fe of the magnetic superexchange interaction of pristine LaFeO_3_ is weakened by the presence of Eu^3+^ or Eu^3+^/Cr^3+^ nearest neighbors in La_0.70_Eu_0.30_FeO_3_ or La_0.70_Eu_0.30_Fe_0.95_Cr_0.05_O_3_, respectively. This is supported by the larger linewidth of S2 for the La_0.70_Eu_0.30_Fe_0.95_Cr_0.05_O_3_ nanoparticles, which, in turn, suggests a random distribution in the numbers of Eu^3+^ and Cr^3+^ cationic neighbors of the Fe^3+^ ions at the A and B sites, respectively^6,34^. The weakening of the S1 and S2 hyperfine magnetic fields in the spectra of the La_0.70_Eu_0.30_Fe_0.95_Cr_0.05_O_3_ nanoparticles implies weakened exchange interactions between Fe^3+^ ions because of the negative super-transferred hyperfine field produced by the half-filled d-orbitals of the Cr^3+^ ions at the sites of the ^57^Fe nuclei^35^. The fact that the spectral intensities at 298 K and 78 K for both sextets, S1 and S2, are the same supports their assignments to the Fe^3+^ environments, as described above.

**Table 3:** The hyperfine parameters derived from fitting the Mӧssbauer spectra recorded at 298 K and 78 K (*between brackets and in italics*) for the LaFeO_3_, La_0.70_Eu_0.30_FeO_3_ and La_0.70_Eu_0.30_Fe_0.95_Cr_0.05_O_3_ nanoparticles.

	Sub-spectrum	δ (mms) ± 0.02	ε (mms) ± 0.02	H_eff_ (T) ± 0.2	Area (%) ± 2
LaFeO_3_	S	0.37(*0.47*)	− 0.04(− *0.03*)	51.5(*55.5*)	100(*100*)
La_0.7_Eu_0.3_FeO_3_	S1	0.37(*0.47*)	− 0.02(− *0.02*)	51.0(*55.3*)	88(*89*)
	S2	0.38 (*0.49*)	− 0.06 (*0.00*)	47.3(*53.1*)	12(*11*)
La_0.7_Eu_0.3_Fe_0.95_Cr_0.05_O_3_	S1	0.37(*0.47*)	− 0.03(− *0.01*)	50.7(*55.2*)	86(*86*)
	S2	0.41(*0.50*)	0.04(− *0.03*)	47.1(*52.6*)	14(*14*)

*S* magnetic sextet, *δ* isomer shift, *ε* quadrupole shift, *H*_*eff*_ hyperfine magnetic field.

### Surface composition: XPS spectral analysis

**Figure 6:**
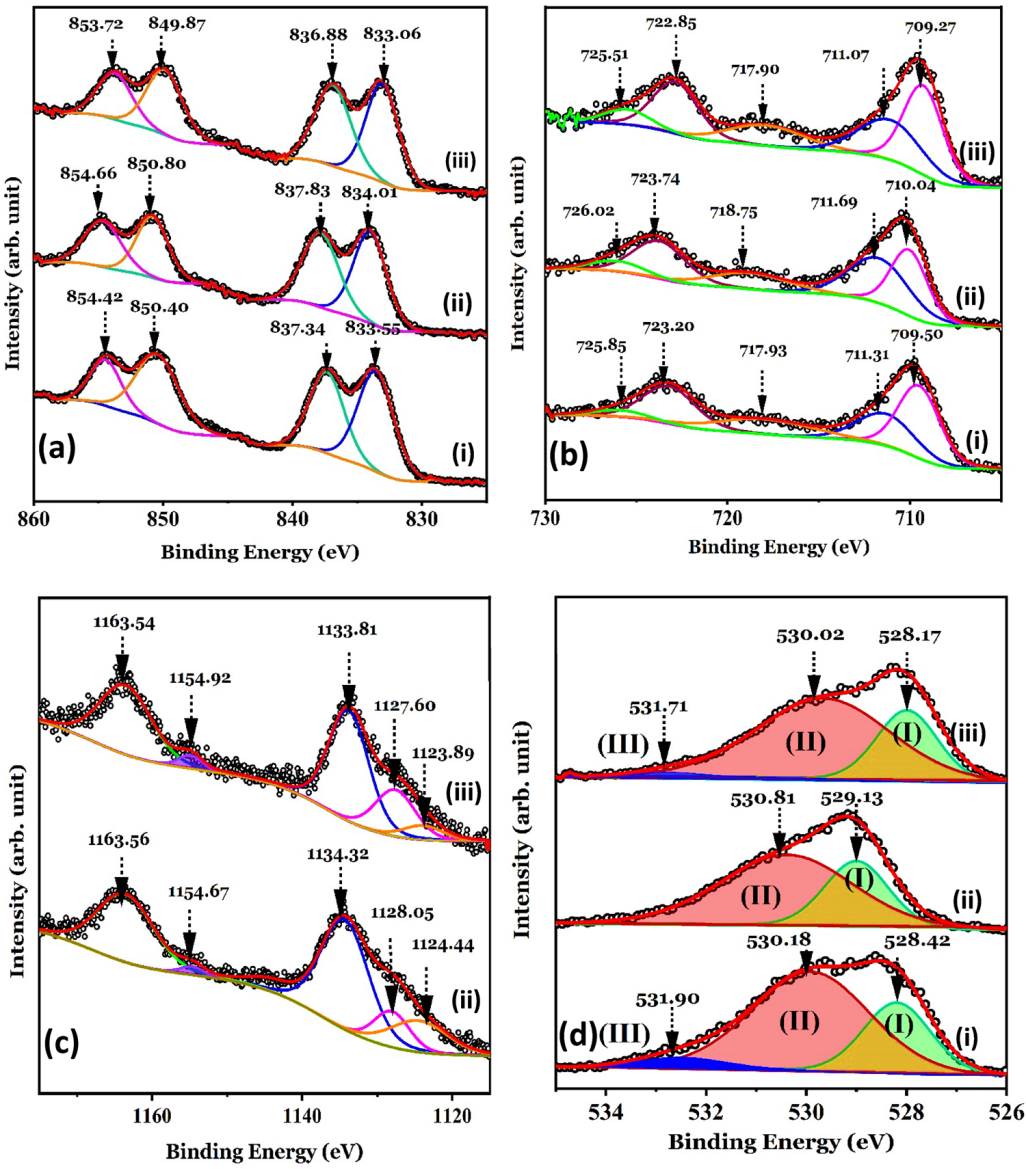
The (**a**) Fe 2p, (**b**) La 3d, (**c**) Eu 3d, and (**d**) O 1s core-level XPS spectra recorded from the (i) LaFeO_3_, (ii) La_0.70_Eu_0.30_FeO_3_, and (iii) La_0.70_Eu_0.30_Fe_0.95_Cr_0.05_O_3_ nanoparticles. The solid lines represent the best fitting of the experimental data. The estimated error for the binding energy values is ± 0.01 eV.

The La 3d, Eu 3d, Fe 2p and O1s XPS spectra of the LaFeO_3_ nanoparticles and their Eu^3+^-doped and Eu^3+^/Cr^3+^ co-doped modifications are shown in Fig. 6. The C 1s XPS peak at 284.6 eV was taken as a reference for all charge shift corrections. The La 3d_3/2_ and La 3d_5/2_ peaks of the LaFeO_3_ sample at the binding energies (BE) 850.4 eV and 833.6 eV (Fig. 6a) are similar to those reported for sol–gel processed LaFeO_3_ nanoparticles^7,36^. Satellite peaks at BE values 854.4 eV and 837.3 eV correspond to the shake-up of the La 3d_3/2_ and La 3d_5/2_ states, respectively, due to electron transfer from the O 2p valence band to empty La 4f states^36^. As these BE values have been reported for both La_2_O_3_ and LaFeO_3_^7,37^, the existence of some unreacted La_2_O_3_ components on the surface of these mechano-synthesized LaFeO_3_ nanoparticles could not be ruled out. Additionally, these BE values may be partly associated with the very small amounts of Fe-doped La_2_O_3_^38^ and/or La-doped α-Fe_2_O_3_^39^ intermediate phases that develop during the reaction, leading to the LaFeO_3_ phase. The BE values of the La 3d_3/2_ and La 3d_5/2_ spectra for the La_0.70_Eu_0.30_FeO_3_ nanoparticles, 850.8 eV and 834.0 eV, and those of the La_0.70_Eu_0.30_Fe_0.95_Cr_0.05_O_3_ nanoparticles, 849.9 eV and 833.1 eV, are slightly higher than those of the LaFeO_3_ nanoparticles. This implies that introducing the more electronegative Eu^3+^ (1.2) for the less electronegative La^3+^ (1.1) shifts the La 3d spectral peaks towards higher BE values because of the lowering of the electron densities^40^. Similarly, the lower BE values for the La 3d spectral peaks of the La_0.70_Eu_0.30_Fe_0.95_Cr_0.05_O_3_ nanoparticles relative to those of the La_0.70_Eu_0.30_FeO_3_ nanoparticles may be attributed to doping with the less electronegative Cr^3+^(1.66) for the more electronegative Fe^3+^ (1.83), which increases the La^3+^ and O^2−^ electron densities and consequently lowers the BE values^40^.

Figure 6b shows the Fe 2p XPS spectra of all the nanoparticles. The asymmetrical Fe 2p_3/2_ peak of the LaFeO_3_ nanoparticles could be decomposed into distinct peaks at BE of 709.5 eV and 711.3 eV corresponding, respectively, to surface Fe^2+^- and Fe^3+^-containing species^7,41^. Previously, we reported a similar reduction of Fe^3+^ to Fe^2+^ on the surface of mechano-synthesized Nd_0.33_Eu_0.67_Cr_*x*_Fe_1−*x*_O_3_ nanoparticles^15^. In addition, the presence of surface Fe^2+^ ions has been reported for other REFeO_3_ compounds in the form of bulk or nanoparticles, such as PrFeO_3_^42^, ErFeO_3_^43^ and Ir-doped YbFeO_3_^44^. In fact, the shake-up satellite peak at a BE value of 717.8 eV, which is located at + 8.3 eV from the Fe 2p_3/2_ peak at 709.5 eV, is typical of satellite signals of ferrous species^41^. The presence of these Fe^2+^ impurities suggests the existence of O^2−^ vacancies as balancing defects. Together, these results imply the formation of an O^2−^ defective perovskite phase of LaFeO_3-δ_ at the surface of the nanoparticles^45^. Hence, we associate the Fe 2p_3/2_ peak at the BE of 709.5 eV to this surface LaFeO_3-δ_ component. The sub-spectral peak at 711.3 eV is almost similar to that of Fe^3+^ ions in α-Fe_2_O_3_ (711.2 eV). Hence, we associate the 711.3 eV peak with the La-doped α-Fe_2_O_3_ intermediate phase implied above by the La 3d XPS spectrum^39^. The Fe 2p_1/2_ spectral component was fitted with two peaks centered at the BE values of 723.2 eV and 725.8 eV which are assigned, respectively, to LaFeO_3-δ_ and either or both of LaFeO_3_ and La-doped α-Fe_2_O_3_^7,36,39^. As is seen in Fig. 6b, the binding energies of the Fe 2p_3/2_ peaks increase for the La_0.70_Eu_0.30_FeO_3_ nanoparticles (710.0, 711.7 eV) relative to the corresponding values of the LaFeO_3_ nanoparticles. This is suggestive that the incorporation of Eu^3+^ in LaFeO_3_ results in lesser O^2−^ vacancies hence more Fe^3+^ ions and, consequently, stronger Fe–O bonds. However, for the La_0.70_Eu_0.30_Fe_0.95_Cr_0.05_O_3_ nanoparticles the binding energies of Fe 2p core-level spectra tend to decrease on the Cr^3+^ substitution. The reason for this is apparently the same as that we argued above for the similar trend in the La 3d spectra following the Cr^3+^ substitution.

Each of the Eu 3d core-level XPS spectra of the Eu^3+^-doped and Eu^3+^/Cr^3+^-co-doped LaFeO_3_ nanoparticles, shown in Fig. 6c, is composed of five peaks. For the La_0.70_Eu_0.30_FeO_3_ sample, the peaks were assigned as follows. The Eu^3+^ doublet at the BE values of 1134.3 eV (Eu 3d_5/2_) and 1163.6 eV (Eu 3d_3/2_) is attributed to a surface Eu^3+^-doped α-Fe_2_O_3_, as is reported elsewhere^46^. The weak Eu^2+^ doublet at the BE values 1124.4 eV (Eu 3d_5/2_) and 1154.7 eV (Eu 3d_3/2_), which is consistent with those reported before for other Eu-containing systems^47^, merits special attention as it appears to influence the nanoparticles’ low temperature magnetic behavior to be discussed in the next section. The presence of Eu^2+^ ions, in systems like the present ones, is attributed to an intermediate valence between the electronic configurations of Eu^3+^ and Eu^2+^ ions^48^. This reduction of Eu^3+^ to Eu^2+^ is implicative of the presence of balancing O^2−^ vacancies on the surface of nanoparticles. Hence, we associate the Eu^2+^ doublet with the weak La_0.7_Eu_0.3_FeO_3-δ_ surface component. The fifth fitted peak in the Eu 3d core level spectrum, at the BE value 1128.1 eV was not, to our knowledge, reported before for any Eu-containing solid. Hence, we assume that it is due to the investigated La_0.70_Eu_0.30_FeO_3_ nanoparticles. Such an assignment appears logical as the Eu 3d core level XPS spectral peak at the close BE value of 1129.0 eV was assigned before to Nd_0.33_Eu_0.67_FeO_3_ nanoparticles^15^.

As is clear from Fig. 6c, the presence of Cr^3+^ in the La_0.70_Eu_0.30_Fe_0.95_Cr_0.05_O_3_ nanoparticles slightly shifts the Eu 3d doublet relative to that of the La_0.70_Eu_0.30_FeO_3_, to the BE values of 1133.8 eV and 1163.5 eV. These values are consistent with those reported for EuCrO_3_ doublets, suggesting the possibility that traces of EuCrO_3_ are present on the surface of La_0.70_Eu_0.30_Fe_0.95_Cr_0.05_O_3_ nanoparticles^25^. In similar lines to those given above, we attribute the Eu 3d core-level XPS peak at the BE value 1123.9 eV and the shake-down satellite at 1154.9 eV, in the spectrum of the La_0.70_Eu_0.30_Fe_0.95_Cr_0.05_O_3_ nanoparticles, to an Eu^2+^-containing surface component that could be La_0.7_Eu_0.3_FeO_3-δ_ and/or La_0.70_Eu_0.30_Fe_0.95_Cr_0.05_O_3-δ_^47^. The fifth peak in the Eu 3d spectrum of the same sample, at the BE value of 1127.6 eV, is negatively shifted and enhanced in intensity relative to the corresponding one in the spectrum of La_0.70_Eu_0.30_FeO_3_. Taken together, these results imply that even a low concentration of the less electronegative Cr^3+^ relative to the substituted Fe^3+^ may significantly influence the surface composition of the mechano-synthesized La_0.70_Eu_0.30_Fe_0.95_Cr_0.05_O_3_ nanoparticles, with the possibility of having surface traces of pure and/or Cr^3+^-doped La_0.70_Eu_0.30_FeO_3-δ_ as well as EuCrO_3_^25^.

The O 1s XPS spectra of the nanoparticles are shown in Fig. 6d. As we have, previously, done with similarly mechano-synthesized rare earth orthoferrites^15^, we resolve the O 1s peak for the LaFeO_3_ nanoparticles into three overlapping peaks. In agreement with the La 3d spectra, the first component of the O 1s peak (I) at a BE value of 528.2 eV is related to perovskite-related LaFeO_3_ and/or moderately Fe-doped La_2_O_3_^7,36,38^. The second component (II) at 529.9 eV is assigned to the α-Fe_2_O_3_ and/or La-doped α-Fe_2_O_3_, both of which are implicated by the Fe 2p spectra discussed above^15,39,41^. The LaFeO_3-δ_ phase, whose presence was inferred from the Fe 2p spectra, is associated with the minor third component of O 1s (III) at the BE value of 532.5 eV^15,41,45^. With Eu^3+^ doping, the BE of the components of the O 1s peak increased to 529.0 eV (I) and 530.4 (II) eV which, in line with the above analysis, we ascribe to La_0.70_Eu_0.30_FeO_3_ and Eu^3+^-doped α-Fe_2_O_3_, respectively^46^. The positive shift of O 1s, in the XPS La_0.70_Eu_0.30_FeO_3_ spectrum, is possibly due to the decrease in the distance between the O^2−^ ion and their doped Eu^3+^ neighbors due to the higher electronegativity of Eu^3+^ relative to that of La^3+^^40^. The absence of the third component (III) in the O 1s XPS spectrum of the La_0.70_Eu_0.30_FeO_3_ sample suggests that doping with Eu^3+^ results in the elimination of the Fe^2+^-containing LaFeO_3-δ_ surface component. With the inclusion of the Cr^3+^ ion, the BE values of the three O 1s XPS peaks decrease slightly to 528.0 eV (I), 529.6 eV (II) and 532.7 eV (III) relative to those of the La_0.70_Eu_0.30_FeO_3_ nanoparticles. Such a reduction is attributed to the lower electronegativity^40^ of Cr^3+^ over Fe^3+^. Based on previous work, and in line with above analysis, the BE values of these O 1s XPS spectral peaks are assigned, respectively to EuCrO_3_, Eu^3+^-doped α-Fe_2_O_3_ and pure and/or Cr^3+^-doped La_0.70_Eu_0.30_FeO_3-δ_ surface components^25^. To summarize, the above XPS data analysis has shown mechano-synthesized Eu^3+^-doped and Eu^3+^/Cr^3+^-co-doped LaFeO_3_ nanoparticles to have complex surface structures, that contain pure and Eu^3+^/Cr^3+^ doped and co-doped LaFeO_3_ phases, O^2^-deficient undoped and Eu^3+^/Cr^3+^-doped LaFeO_3_ phases, traces of undoped or doped initial reactants. Such complexity is expected on the surfaces of nanoparticles prepared by mechanical milling and subsequent sintering^15,25^.

### The magnetic properties

**Figure 7:**
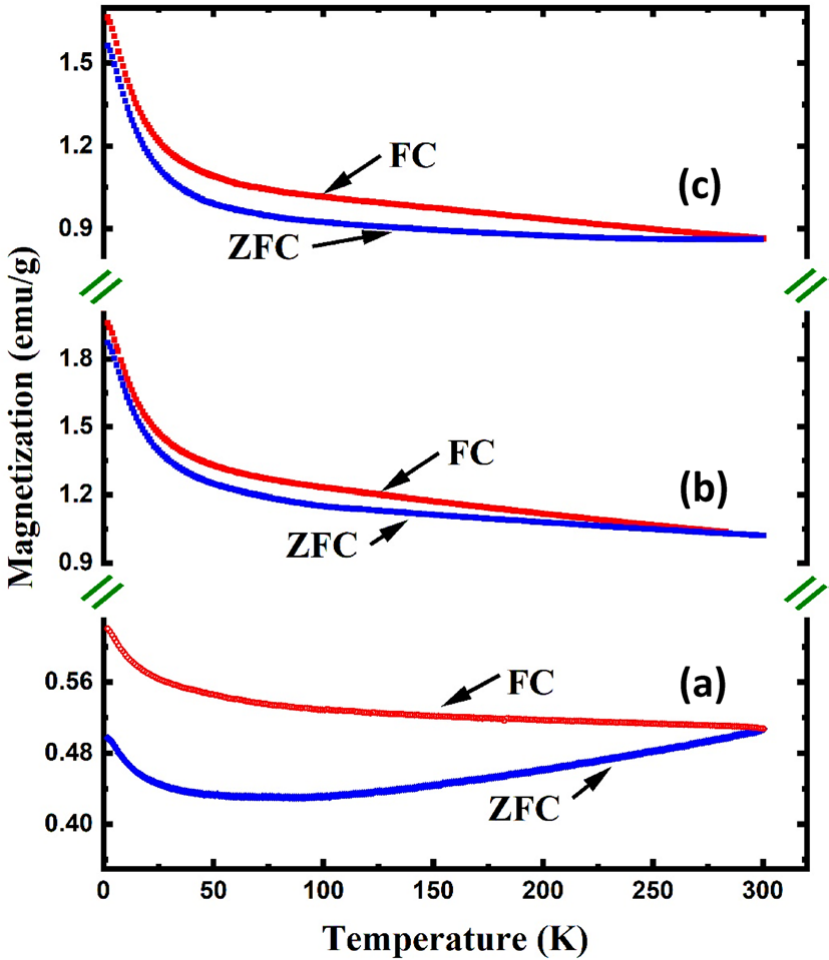
The temperature dependence of the FC and ZFC magnetization of the (**a**) LaFeO_3_, (**b**) La_0.70_Eu_0.30_FeO_3_, and (**c**) La_0.70_Eu_0.30_Fe_0.95_Cr_0.05_O_3_ nanoparticles under an applied field of 50 kOe.

Figure 7 shows the temperature dependence of the FC and ZFC magnetization in the range of 2–300 K under an applied field of 50 kOe for LaFeO_3_, Eu^3+^-doped, and Eu^3+^/Cr^3+^-co-doped LaFeO_3_ nanoparticles. As expected for a magnetically ordered system, the FC magnetization curves for all samples exhibited similar temperature dependencies to their Mössbauer magnetic hyperfine fields, decreasing with increasing temperature. The wider bifurcation observed for the LaFeO_3_ nanoparticles reveals stronger thermomagnetic irreversibility in comparison to the Eu^3+^-doped and Eu^3+^/Cr^3+^-co-doped nanoparticles^13^. The ZFC magnetization of the LaFeO_3_ nanoparticles initially decreased with decreasing temperature to a minimum and then increased without exhibiting a clear maximum below the bifurcation point^49^. Based on the Mössbauer data, this could be explained in terms of the core/shell model, as was done by other^1,13,16,49^ researchers. While the core is AFM in nature, the spins of the glass-like shells assume an FM ordering owing to the AFM spin canting at the cores in addition to the field applied during the measurement. The magnetization enhancement to values similar to those previously reported for LaFeO_3_ nanoparticles at very low temperatures could result from a local spin order that develops at the interface between the canted AFM cores and FM shells as a result of strong spin coupling^9,13,50^. With a gradual increase in temperature, the interfacial exchange coupling weakens, while the core spins resist alignment even by a field as high as 50 kOe^50^. Thus, the core–shell AFM-FM coupling requires higher thermal agitation and/or a higher applied field to be weakened, as manifested by the development of the spin-glass-like behavior at ~ 75 K (Fig. 7). This may explain why spin-glass-like behavior was not detected by zero-field Mössbauer measurements at 78 K. At temperatures higher than 75 K, the spins of the FM shell start to reverse their orientation in the direction of the applied field, and concomitantly, the AFM coupling at the cores starts to decay. Consequently, the ZFC magnetization increased with a further increase in temperature, as observed^9,10^. Similar behavior was not observed in the FC curve of the same sample because the surface spins were readily aligned in the field direction, resulting in an apparently higher magnetization relative to that obtained in the ZFC case (Fig. 7).

**Figure 8:**
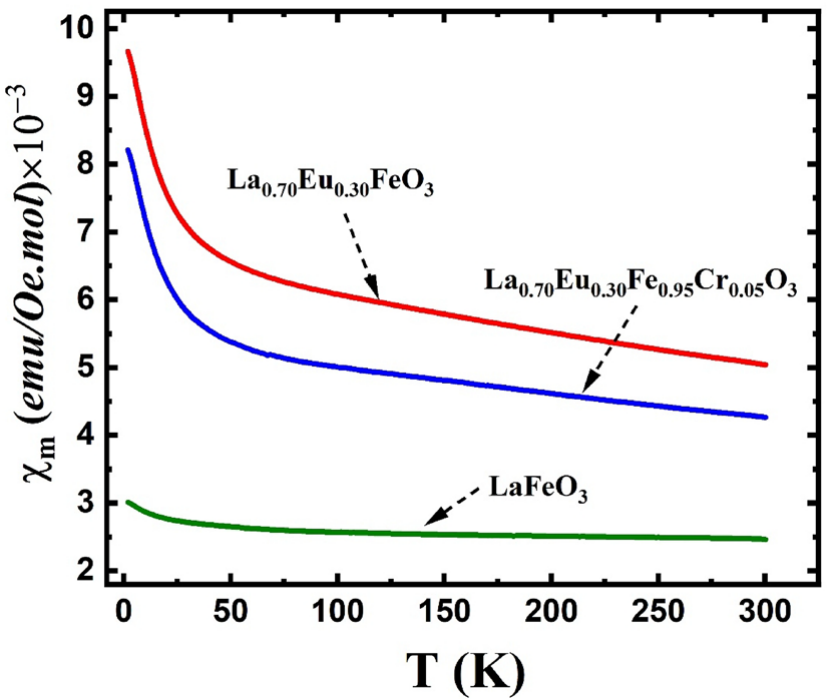
Temperature dependence of magnetic molar susceptibility for the LaFeO_3_, La_0.70_Eu_0.30_FeO_3_ and La_0.70_Eu_0.30_Fe_0.95_Cr_0.05_O_3_ nanoparticles under an applied field of 50 kOe.

**Figure 9:**
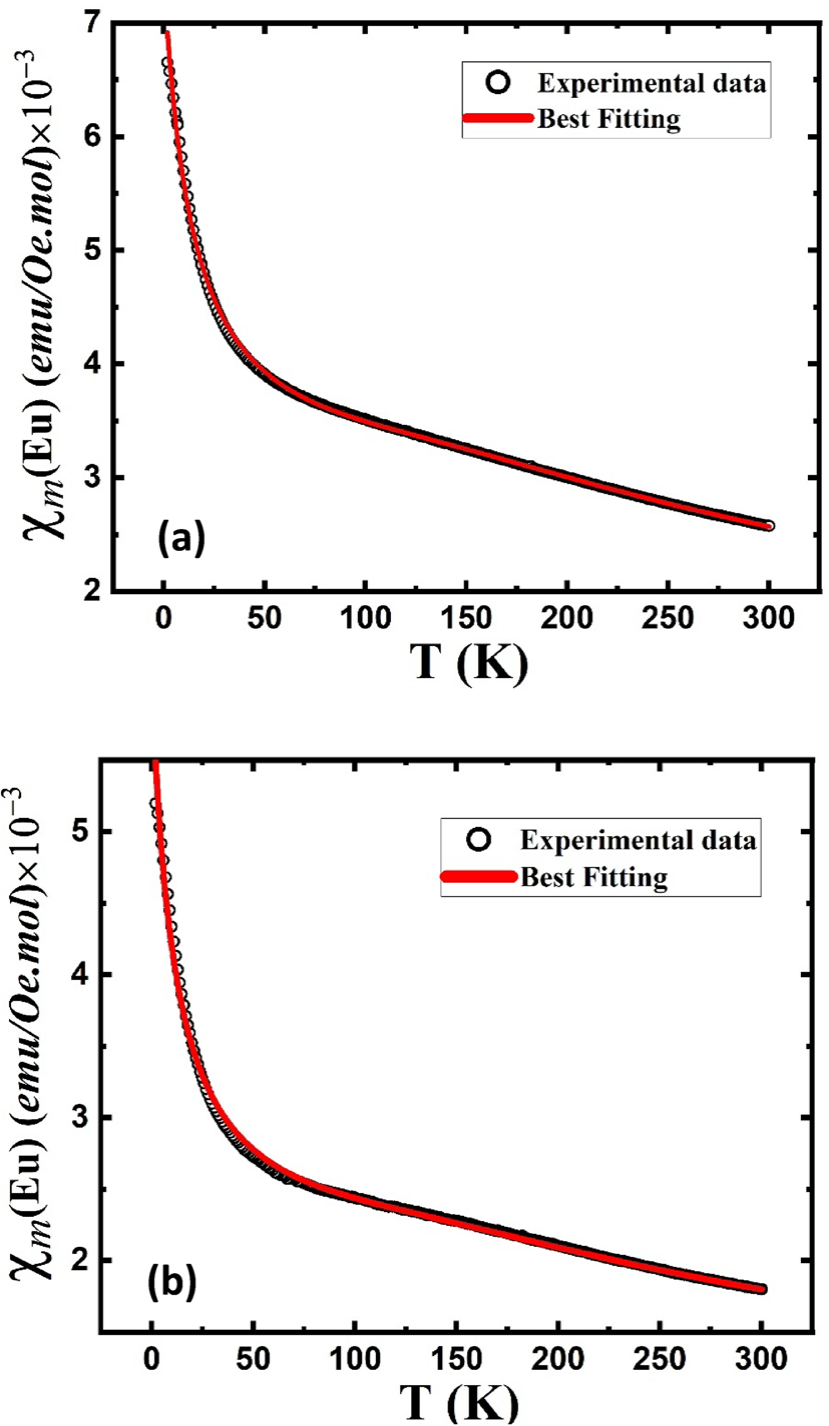
Temperature dependence of the FC Eu-related molar susceptibility $\chi_{m}$ (Eu) for the (**a**) La_0.70_Eu_0.30_FeO_3_ and (**b**) La_0.70_Eu_0.30_Fe_0.95_Cr_0.05_O_3_ nanoparticles, obtained by subtracting the molar susceptibility contribution of the LaFeO_3_ nanoparticles from that of the La_0.70_Eu_0.30_FeO_3_ and La_0.70_Eu_0.30_FeO_3_ ones, respectively, measured in an applied field of 50 kOe. The solid line represents the best fitting line based on Eqs. (1) and (2).

As shown in Fig. 7, with decreasing temperature, the ZFC and FC magnetization values for the La_0.70_Eu_0.30_FeO_3_ and La_0.70_Eu_0.30_Fe_0.95_Cr_0.05_O_3_ nanoparticles increased throughout the scanned temperature range. The higher magnetization values for both materials relative to those of the pristine LaFeO_3_ sample are evidently linked to the introduction of paramagnetic Eu ions as partial substituents for diamagnetic La^3+^. A comparison of the FC molar susceptibility (*χ*_*m*_) values of the three materials, as depicted in Fig. 8, reveals that the magnetic contribution of the Fe^3+^ ions is barely detectable in LaFeO_3_ compared to that of the Eu ions in the other two compounds. To determine the contribution of Eu^3+^ and XPS-detected minority surface Eu^2+^ ions to the total susceptibility of both the Eu-doped samples, hereafter referred to as* χ*_*m*_(Eu), we subtracted *χ*_*m*_ of the LaFeO_3_ from that of either. The rationale here is to exclude the magnetic effect of the Fe^3+^ sublattice, which is similar in all three compounds. Each of the obtained *χ*_*m*_(Eu) curves, shown in Fig. 9a (La_0.70_Eu_0.30_FeO_3_) and 9-b (La_0.70_Eu_0.30_Fe_0.95_Cr_0.05_O_3_), may be divided into two temperature regions, viz*.* 2–20 K and 20–300 K. In the first low temperature region, the observed upward ascending trend of *χ*_*m*_(Eu) below 20 K is implicative of a Curie–Weiss type paramagnetism that is solely associated with the minority Eu^2+^ ions having an electronic configuration of [Xe] 4f^7^ and a theoretical effective magnetic moment μ_*eff*_ of 7.94 μ_B_ similar^48^ to that of Gd^3+^. In the second region, the behavior of* χ*_*m*_(Eu) was attributed *mainly* to the van Vleck paramagnetism of the Eu^3+^ excited states^48^. According to the Russell–Saunders formalism, Eu^3+^ ([Xe] 4f^6^) has a total angular momentum (*J*) of zero and, hence, a nonmagnetic ground state (^7^F_0_)^53^. Nevertheless, Eu^3+^ has a spin–orbit interaction given by (λ*L.S*) with *L* and *S* orbital and spin angular momenta, respectively, and (λ) is the spin–orbit coupling constant^47,48,50^. In the framework of the van Vleck theory, the fact that the differences between the ground state and the low-lying excited states ^7^F_*J*_ of the Eu^3+^ ion are of the order of *k*_B_*T* at room temperature (207 cm^−1^) results in the Eu^3+^ ions contributing a temperature-dependent term ($\chi$) to the paramagnetic susceptibility, whose value depends on λ^48^. For instance, while for insufficiently large values of λ relative to (207 cm^−1^), a non-negligible magnetic moment might be observed at low temperatures, the Eu^3+^ ions contribute a minute van Vleck temperature-independent paramagnetic term ($\chi_{0}$) that is linked to the ^7^F_0_ state^48^. Based on the above, the *χ*_*m*_(Eu) curves in Fig. 9a,b were not amenable to satisfactory fits by separately applying a Curie–Weiss type equation to the low-temperature data and a van Vleck type equation fit to the data in the ~ 20–300 K temperature range. However, the best fits of each of the *χ*_*m*_(Eu) curves in the entire temperature range of 2–300 K were obtained by considering both the temperature-independent and-dependent Eu^3+^ van Vleck terms in addition to the Eu^2+^ Curie–Weiss type paramagnetic term using the following equation^48^:

1 $$ \chi_{m } (Eu) = \chi_{o} + n\chi_{m } (Eu^{2 + } ) + (1 - n)\chi_{m } (Eu^{3 + } ) $$

where *n* is the relative Eu^2+^ contribution to the total *χ*_*m*_(Eu) in the temperature range of the measurement. By limiting ourselves to the first three excited states of Eu^3+^, Eq. (1) can be rewritten as follows:

2 $$ \begin{aligned} \chi_{m} (Eu) & = \chi_{0} + n \times \frac{C}{{T - \theta_{W} }} + (1 - n) \times \frac{{N_{A} \mu_{B}^{2} }}{{3k_{B} T}} \\ & \quad \times \frac{{(24/a) + (13.5 - 1.5)e^{ - a} + (67.5 - 2.5)e^{ - 3a} + (189 - 3.5)e^{ - 6a} }}{{1 + 3e^{ - a} + 5e^{ - 3a} + 7e^{ - 6a} }} \\ \end{aligned} $$

where *N*_*A*_ is Avogadro’s constant, *θ*_*W*_ is the Weiss temperature, *a* = *λ/k*_B_*T*. The other symbols have the usual meanings. This has yielded for the La_0.70_Eu_0.30_FeO_3_ nanoparticles the values of μ_eff_ = 0.72μ_B_, *θ*_*W*_ ~ − 14 K, *n* = 0.58, λ = 363 cm^−1^ and $\chi_{0}$ = 5.3 × 10^−5^ emu/Oe. mol. Similar values were obtained for the La_0.70_Eu_0.30_Fe_0.95_Cr_0.05_O_3_ except for *n* and *θ*_*W*_, which were found to be = 0.70 and ~ − 11 K, respectively. Starting with the low-temperature upturn in *χ*_*m*_(Eu) that we ascribed to Eu^2+^ spins, it is evident that the negative values of *θ*_*W*_ imply the presence of AFM interactions among the Eu^2+^ spins. However, the obtained *θ*_*W*_ values for both compounds reflect the nature of this weak AFM interaction. The value of μ_eff_ = 0.72μ_B_/Eu^2+^ is very small when compared to the corresponding theoretical value for a free Eu^2+^ ion (7.94 µB)^50^. This could possibly reflect the fact that the Eu ions in both materials were in a valence fluctuation state with no localized magnetic moments. We now turn to the remaining part of the scanned temperature range, where the obtained value of λ suggests that the energy interval between the ground state (^7^F_0_) and first excited (^7^F_1_) state for Eu^3+^ (4f^6^) ions, in both compounds, primarily influences the paramagnetic susceptibility above 521 K. This, in turn, explains the weak relative contribution of the Eu^3+^ van Vleck temperature-dependent susceptibility to the total *χ*_*m*_(Eu), viz*.* 0.42 (La_0.70_Eu_0.30_FeO_3_) and 0.30 (La_0.70_Eu_0.30_Fe_0.95_Cr_0.05_O_3_) in the 20–300 K temperature range. The fact that these results are slightly different from those reported for other Eu-containing oxide systems^48^ could possibly be ascribed to the high applied field (H = 50 kOe), crystal field anisotropy of the distorted crystal structure, and screening effect, as indicated by the increase in the Mössbauer isomer shift of the S2 sextet (Table 3).

**Figure 10:**
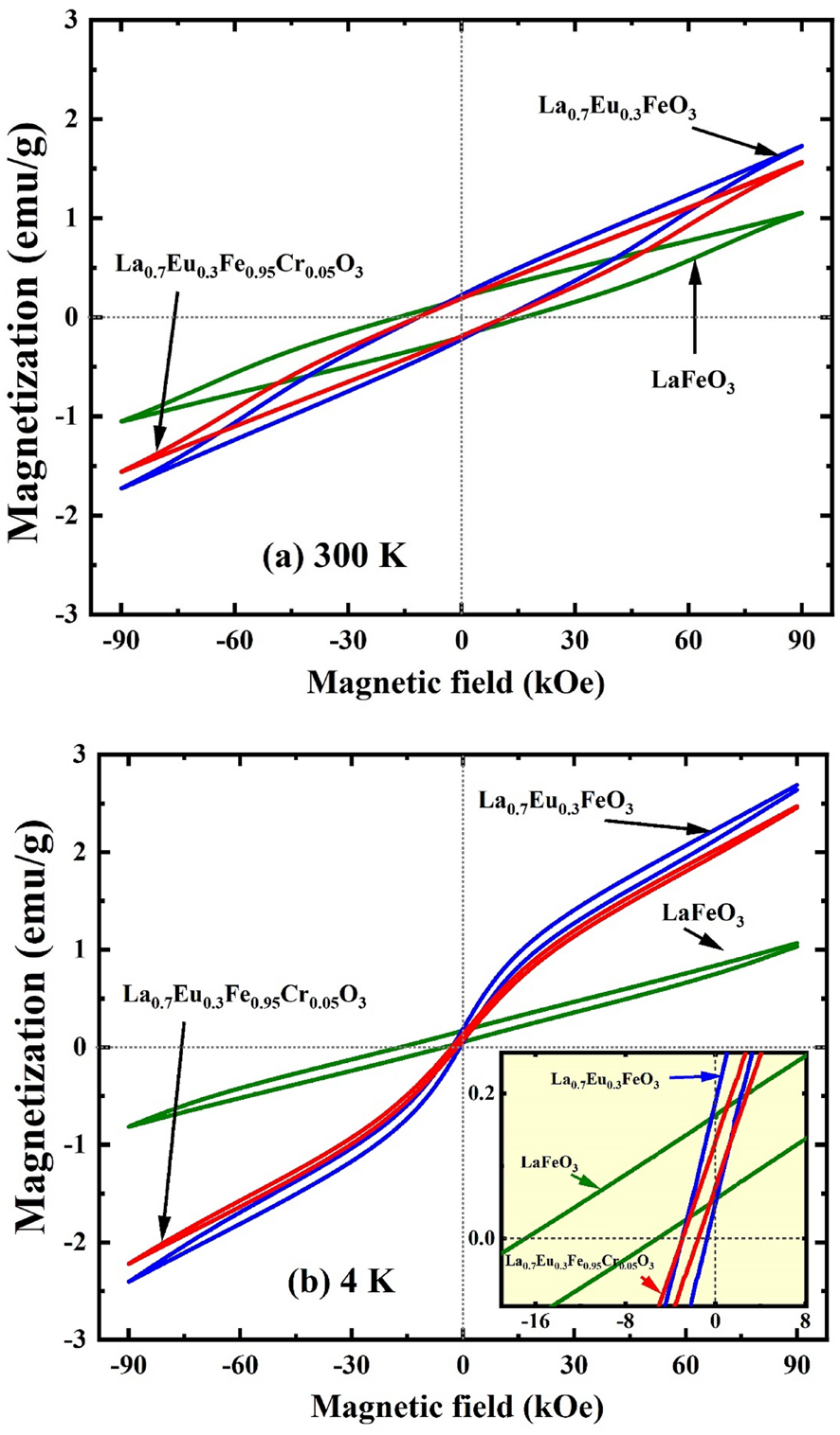
The hysteresis loops (M–H curves) of the LaFeO_3_, La_0.70_Eu_0.30_FeO_3_, and La_0.70_Eu_0.30_Fe_0.95_Cr_0.05_O_3_ nanoparticles at (**a**) 300 K and (**b**) 4 K. The inset shows a zoom-in image of the same loops to display the non-zero values of both coercivity and remnant magnetization at 2 K.

Figure 10 shows that the magnetization values of the La_0.70_Eu_0.30_Fe_0.95_Cr_0.05_O_3_ nanoparticles were lower than those of the La_0.70_Eu_0.30_FeO_3_ nanoparticles. This is presumably due to the substitution of the weaker magnetic Cr^3+^ ion (µ = 3 µ_B_) for the stronger Fe^3+^ ion (µ = 5 µ_B_), which weakens the AFM coupling relative to that of the La_0.70_Eu_0.30_FeO_3_ nanoparticles^13,51^. The slight increase in the bifurcation of the La_0.70_Eu_0.30_Fe_0.95_Cr_0.05_O_3_ nanoparticles relative to that of the La_0.70_Eu_0.30_FeO_3_ nanoparticles can be attributed to an increased interface anisotropy caused by co-doping with both Eu^3+^ and Cr^3+^ ions. Considering the limited temperature range used for the FC and ZFC scans, we conclude this section by qualitatively commenting on the irreversibility temperature (T_irr_) of the nanoparticles (Fig. 7). The value of T_irr_ for La_0.70_Eu_0.30_FeO_3_, ~ 287 K, is lower than the corresponding values for the undoped and Eu^3+^/Cr^3+^-co-doped LaFeO_3_ counterparts, both of which could not be determined firmly in the scanned temperature range. The reduction in T_irr_ for the La_0.70_Eu_0.30_FeO_3_ sample relative to that of the LaFeO_3_ sample can be explained using the argument given above for the enhancement of the ZFC magnetization values to approach those of the FC magnetization owing to doping with Eu^3+^. Similarly, the higher T_irr_ of the La_0.70_Eu_0.30_Fe_0.95_Cr_0.05_O_3_ nanoparticles relative to that of the La_0.70_Eu_0.30_FeO_3_ nanoparticles is associated with the diminution of the ZFC magnetization values relative to those of the FC, following the introduction of Cr^3+^ ions.

A final comment in this part goes for the high applied field of 50 kOe used in the FC and ZFC measurements of Fig. 7 and the discussion that followed. This is exactly the same applied field value, deemed necessary to saturate the canted ferromagnetic moments of Fe^3+^, by Ahmadvand et al.^49^ in investigating the exchange bias effect in the pristine LaFeO_3_. This is why it was easy to exclude the contribution of the Fe^3+^ moments in the above calculations of molar susceptibility. This spin canting arises from the disturbing effect of the crystalline field on the much stronger exchange field, as evidenced by hysteresis loops. Fitting the thermal-dependent magnetization at high field (1.5 kOe ≤ H ≤ 50 kOe) and low temperatures with the Curie–Weiss law, the saturation magnetization almost exclusively represents the rare earth moment, as found in^52^.

To gain insight into the static features of the magnetic behavior in the three samples, temperature-dependent DC magnetization was performed under an applied field of 100 Oe, as shown in Fig. S1. One notices two distinct glassy states to emerge at ~ 93 K and ~ 90 K for the LaFeO_3_ nanoparticles, that shift slightly to ~ 92 K and ~ 84 K for both the Eu^3+^ doped and Eu^3+^/Cr^3+^ co-doped variants. This behavior may be linked to the magnetic anisotropy associated with the formation of short-range ordered ferromagnetic clusters and/or a strain-related spin reorientation process for the surface Fe^3+^ cations^53^.

The ZFC hysteresis loops of the LaFeO_3_, La_0.70_Eu_0.30_FeO_3_ and La_0.70_Eu_0.30_Fe_0.95_Cr_0.05_O_3_ nanoparticles were measured at 300 K, 200 K, 100 K and 4 K under an applied field (H) varying between − 9 T and 9 T. Only the loops measured at 300 K and 4 K are shown in Fig. 10. Generally, the loops exhibit a superposition of a dominant contribution ascribed to the AFM core and an FM contribution stemming from both the uncompensated spin-glass shell and canted AFM spins at the core^2,11,13,16,19^. The values inferred from Fig. 10 for some of the magnetic parameters are listed in Table 4^2^. The fact that a field as high as 9 T is not sufficient to saturate *M* at all temperatures indicates the dominant AFM behavior of the cores. Moreover, the hysteresis loops of the Eu-containing nanoparticles at 4 K, as compared to the pristine sample, exhibit a linear non-compensated paramagnetic contribution as well as a weak ferromagnetic behavior, as shown in the inset of Fig. 10b. The paramagnetic behavior is obviously due to the Curie Weiss-like behavior that was attributed to the surface Eu^2+^ ions at low temperatures in the preceding section. The small remnant magnetization (FM contribution) is due to the canted tendency of the FeO_6_ octahedra, which affects the superexchange of neighboring Fe t_2g_ electrons through Dzyaloshinskii–Moriya (DM) interactions^19,50^. The values of the instantaneous magnetization in the different M-H isotherms follow similar trends to those obtained for the FC magnetization of the nanoparticles (Fig. 8), such that La_0.70_Eu_0.30_FeO_3_ has the highest *M* values and LaFeO_3_ has the lowest magnetoelectric coupling in these multiferroic nanoparticles, which reduces the effective magnetic anisotropy^54^.

**Table 4:** The maximum magnetization (*M*_*max*_), coercivity (*H*_*c*_), and exchange bias (*EB*) of the mechano-synthesized LaFeO_3_, La_0.70_Eu_0.30_FeO_3_ and La_0.70_Eu_0.30_Fe_0.95_Cr_0.05_O_3_ nanoparticles at 300 K, 200 K, 100 K and 4 K. Estimated errors are given in parentheses.

		*M*_*max*_ (emu/g) [$\mu_{B}$/f.u.]	|*H*_*c*_| (kOe)	|*EB*| (Oe)
LaFeO_3_	300 K	1.055(7) [0.05]	16.580(3)	216.650(3)
La_0.70_Eu_0.30_FeO_3_		1.733(3) [0.08]	11.361(1)	77.900(1)
La_0.70_Eu_0.30_Fe_0.95_Cr_0.05_O_3_		1.566(7) [0.07]	11.538(1)	98.900(1)
LaFeO_3_	200 K	1.043(9) [0.05]	17.028(8)	5061.945(8)
La_0.70_Eu_0.30_FeO_3_		1.867(6) [0.08]	11.595(5)	2086.025(5)
La_0.70_Eu_0.30_Fe_0.95_Cr_0.05_O_3_		1.708(6) [0.08]	9.470(7)	3239.720(7)
LaFeO_3_	100 K	1.021(7) [0.04]	13.365(8)	8782.325(8)
La_0.70_Eu_0.30_FeO_3_		2.027(3) [0.09]	9.218(9)	3775.390(9)
La_0.70_Eu_0.30_Fe_0.95_Cr_0.05_O_3_		1.856(4) [0.08]	5.343(9)	5008.875(9)
LaFeO_3_	4 K	1.038(5) [0.05]	5.051(5)	8523.250(5)
La_0.70_Eu_0.30_FeO_3_		2.642(6) [0.12]	1.109(7)	1838.700(7)
La_0.70_Eu_0.30_Fe_0.95_Cr_0.05_O_3_		2.457(7) [0.11]	0.700(1)	2221.600(1)

**Figure 11:**
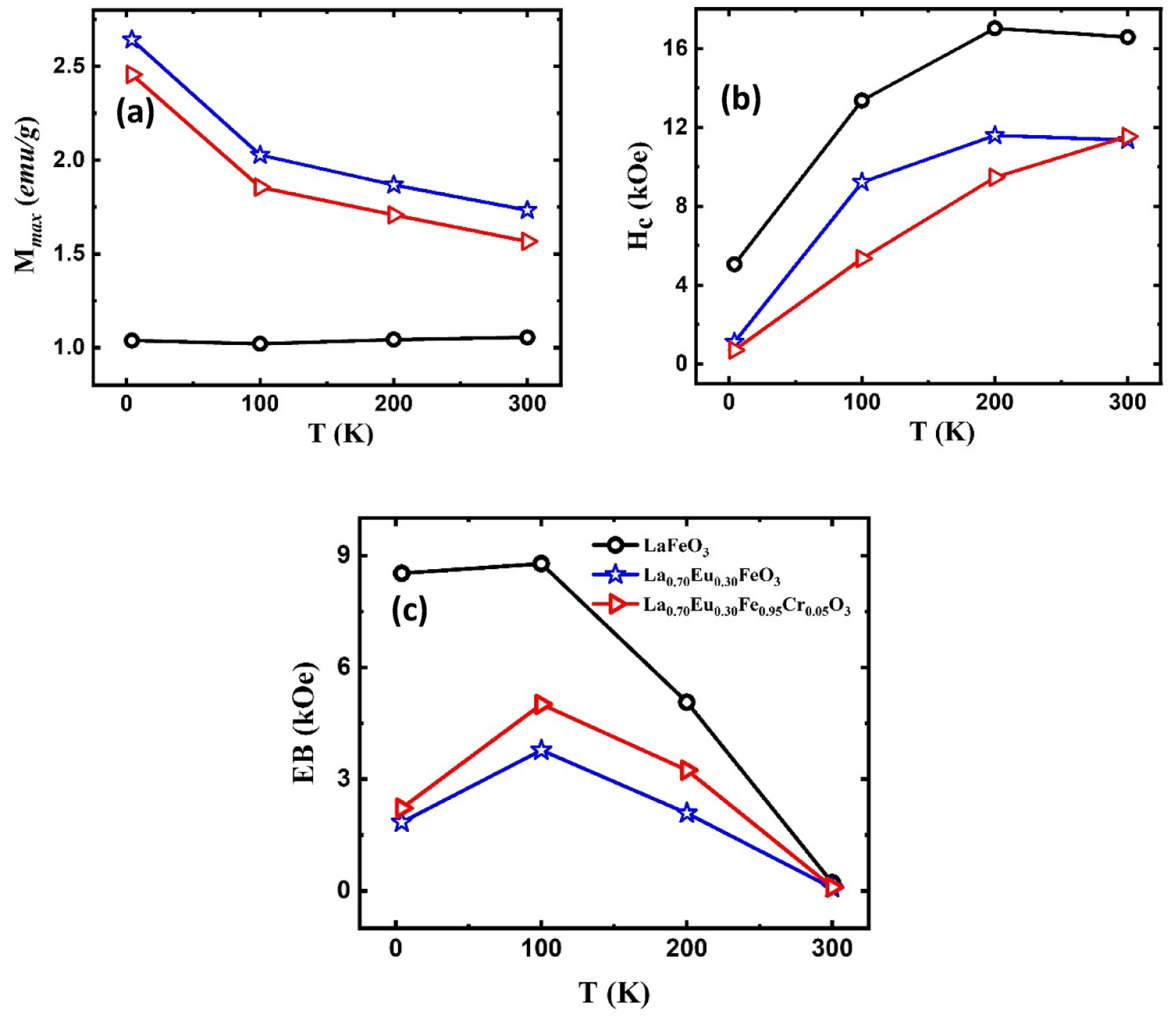
The thermal variation of (**a**) the maximum magnetization (*M*_*max*_), (**b**) coercivity (*H*_*c*_) and (**c**) exchange bias field (*EB*) for the LaFeO_3_, La_0.70_Eu_0.30_FeO_3_ and La_0.70_Eu_0.30_Fe_0.95_Cr_0.05_O_3_ nanoparticles. The symbols in all figures are as shown in the legend of (**c**).

While the values of *M*_*max*_ for the doped nanoparticles decreased with increasing temperature, as expected (Fig. 11a), for the LaFeO_3_ nanoparticles, *M*_*max*_ was almost constant in all hysteresis isotherms. This is explicable in terms of the weakening of the exchange coupling of the spins at the AFM/FM core/shell interface, as suggested above, to enhance the ZFC magnetization of the same nanoparticles with temperature. Consequently, under the same maximum applied field (9 T), *M*_*max*_ was expected to be constant. The values derived from Fig. 10 for the coercivity *H*_*c*_ (= (*H*_*c1*_ − *H*_*c2*_)/2), where *H*_*c1*_ and *H*_*c2*_ are the coercive fields to the right and left of *H* = 0 values, are given in Table 4. Clearly, the doped samples had smaller *H*_*c*_ values relative to the LaFeO_3_ nanoparticles. This may be partly explained in terms of the Stoner-Wohlfarth model, which relates the reduction in *H*_*c*_ to a corresponding reduction in magnetocrystalline anisotropy^55^. The smaller average particle sizes of the doped nanoparticles relative to those of the LaFeO_3_ ones enhance the influence of the uncompensated surface spins that become susceptible to easy flipping by the applied field, thereby reducing *H*_*c*_. It is interesting to note from the temperature dependence of *H*_*c*_ in Fig. 11b that, rather than becoming magnetically harder with decreasing temperature, the mechano-synthesized LaFeO_3_ and La_0.70_Eu_0.30_FeO_3_ nanoparticles softened as the temperature decreased from 200 to 4 K. For the La_0.70_Eu_0.30_Fe_0.95_Cr_0.05_O_3_ nanoparticles, this range extends to 300–4 K. Such an unusual thermal dependence of *H*_*c*_ has been reported before for sol–gel prepared LaFeO_3_ nanoparticles, where the coercivity was found to decrease by *ca. *15% upon cooling from 220 to 5 K^49^. In the present case, *H*_*c*_ for the LaFeO_3_ and La_0.70_Eu_0.30_FeO_3_ has been significantly reduced by *ca.* 70% and 90%, respectively, between 200 and 4 K. For the La_0.70_Eu_0.30_Fe_0.95_Cr_0.05_O_3_, it has dropped by ~ 95% as the temperature was changed from 300 to 4 K. This anomalous magnetic softening may be attributed to the competition between the magnetic anisotropy and magnetoelectric coupling in these multiferroic nanoparticles, which reduces the effective magnetic anisotropy^56^.

The negative *H*_*c*_ shifts in the M-H loops for all the samples (Fig. 10) imply the presence of an exchange bias (*EB*) anisotropy. This is further evidence that the nanoparticles are composed of AFM cores and FM-like shells because *EB* is known to develop owing to the competition between the exchange and Zeeman energies at the interface of both magnetic structures^49^. The values of the EB fields, *EB* (= (*H*_*c1*_ + *H*_*c2*_)/2), for the different nanoparticles are listed in Table 4, and their thermal variation is depicted in Fig. 11c. It can be observed that the *EB* fields increased, reaching a maximum value at 100 K, and then decreased with increasing temperature. The temperature dependence of the *EB* fields can be related to the thermal activation of the spin reversal in the AFM cores of the particles, which plays a significant role in inducing EB anisotropy in the FM/AFM interfaces of the nanoparticles^13,52^. Initially, the applied field induced a preferred spin alignment in some nanoparticles. When the field direction is reversed, the spins in some nanoparticles, particularly those with large volumes, may not gain sufficient thermal energy to reverse direction. Consequently, the core spins of these nanoparticles did not contribute to the hysteresis loop during the entire cycle, thereby inducing spontaneous *EB* anisotropy^13^. This may explain why the LaFeO_3_ nanoparticles, which had the largest particle size and size distribution, as shown in Fig. 2, had the highest *EB* fields relative to the doped nanoparticles. Throughout the temperature range investigated, the lowest *EB* values were recorded for La_0.70_Eu_0.30_FeO_3_ nanoparticles. As the *EB* field values in Fig. 11c reflect the strength of the exchange magnetic coupling, it is evident that doping with Eu^3+^ ions weakens the coupling in the AFM cores. The sharp suppression of *EB* in the doped nanoparticles at 4 K may demonstrate the robust low-temperature impact of the relatively small amount of surface Eu^2+^ ions, as indicated by the susceptibility analysis above. The slight increase in the *EB* field values for the La_0.70_Eu_0.30_Fe_0.95_Cr_0.05_O_3_ nanoparticles relative to those of La_0.70_Eu_0.30_FeO_3_ may be attributed to the distribution of the dopant Cr^3+^ ions in the La_0.70_Eu_0.30_FeO_3_ ionic matrix, where the formation of the Cr^3+^–O^2−^–Cr^3+^ AFM superexchange interaction is more favorable^13^ than that of Fe^3+^–O^2−^–Cr^3+^. This in turn strengthens the overall exchange coupling within the AFM cores. As a final comment in this section, we note that the present mechano-synthesized LaFeO_3_ nanoparticles show higher *EB* fields than those of similar sizes prepared using other synthesis routes. For example, at 300 K, the *EB* value was ~ 217 Oe relative to 139 Oe for LaFeO_3_ nanoparticles prepared via the sol–gel route^57^ and ~ 8.523 kOe (4 K) relative to 1.205 kOe (5 K)^49^. Apparently, this is related to the complex surface composition induced by the mechano-synthesis route, as discussed in section “Surface composition: XPS spectral analysis”, which has no counterpart in the case of LaFeO_3_ nanoparticles prepared using other techniques.

## Conclusion

Single-phase perovskite-related LaFeO_3_, La_0.70_Eu_0.30_FeO_3_ and La_0.70_Eu_0.3_Fe_0.95_Cr_0.05_O_3_ nanoparticles (20–50 nm) were mechano-synthesized at 600 °C (10 h) for the pure and 700 °C (10 h) for the two variants. These temperatures are substantially lower by ~ 600–700 °C than those at which the corresponding bulk materials are traditionally synthesized using solid-state methods. Upon Eu^3+^ doping and Eu^3+^/Cr^3+^ co-doping, the perovskite-related orthorhombic LaFeO_3_ structure undergoes a transition from the usual *O*′*-type* distorted orthorhombic structure of orthoferrites to the *O-type* structure. Eu^3+^ ions preferentially occupy the A-sites in the crystal structure of LaFeO_3_, whereas Cr^3+^ ions are randomly distributed over the octahedral B-sites, and the bond angles and lengths are sensitive to doping and co-doping. Both ^57^Fe Mӧssbauer and magnetic measurements suggested that the nanoparticles were composed of dominant canted AFM cores and FM shells because of uncompensated surface spins, and no superparamagnetism was observed. The increase in ZFC magnetization of the LaFeO_3_ nanoparticles at relatively low temperatures is attributed to the resistance of the core spins; hence, the local spin order that develops at the interface between the canted AFM cores and FM shells is to be aligned by the applied magnetic field. The magnetization of the nanoparticles was sensitive to the presence of Eu^3+^ and Cr^3+^ ions. The La_0.70_Eu_0.30_FeO_3_ and La_0.70_Eu_0.30_Fe_0.95_Cr_0.05_O_3_ nanoparticles were found to display Eu^3+^ van-Vleck-type paramagnetic molar susceptibility with a spin–orbit coupling constant of 363 cm^−1^ in the ~ 20–300 K range and a Curie–Weiss like behavior below 20 K due to the minority of the surface Eu^2+^ ions. The *θ*_*W*_ and μ_eff_ values obtained at low temperatures for the Eu-doped nanoparticles suggest a weak AFM interaction between the Eu^2+^ ions and the Eu^2+^/Eu^3+^ valence state fluctuations, hence the absence of localized Eu magnetic moments. All nanoparticles exhibited anomalous magnetic softening with decreasing temperature, which is referred to as the competition between magnetic anisotropy and magnetoelectric coupling. The nanoparticles reveal temperature-dependent dopant-sensitive exchange bias fields that manifest their AFM/FM core–shell nature, reflecting the thermal activation of the spin reversal at their cores.

## Supplementary Information

Supplementary Figure S1.

## Data Availability

The datasets used and/or analyzed during the current study available from the corresponding author on reasonable request.
