# Author Correction: Core and surface structure and magnetic properties of mechano-synthesized LaFeO_3_ nanoparticles and their Eu^3+^ -doped and Eu^3+^/Cr^3+^ -co-doped variants

**DOI:** 10.1038/s41598-024-72330-1

**Published:** 2024-09-12

**Authors:** R. T. Al-Mamari, H. M. Widatallah, M. E. Elzain, A. M. Gismelseed, A. D. Al-Rawas, S. H. Al-Harthi, M. T. Z. Myint, N. Al-Saqri, M. Al-Abri

**Affiliations:** 1 Physics Department, Sultan Qaboos University, Al-Khoudh, P.O. Box 36, 123 Muscat, Oman; 2 Nanotechnology Research Center, Sultan Qaboos University, Al-Khoudh, P.O. Box 17, 123 Muscat, Oman

Correction to: *Scientific Reports* 10.1038/s41598-024-65757-z, published online 26 June 2024.

The original version of this Article contained an error in the abstract, where the first sentence was incorrect.

As a result, in the Abstract,

“The core and surface structure, and magnetism of mechano-synthesized LaFeO_3_ nanoparticles (30–40 nm), Eu^3+^-doped (La_0.70_Eu_0.30_FeO_3_), and Eu^3+^/Cr^3+^-co-doped (La_0.70_Eu_0.30_Fe_0.95_Cr_0.05_O_3_) are reported.”

now reads:

“The core and surface structure and magnetic properties of mechano-synthesized LaFeO_3_ nanoparticles (30–40 nm), their Eu^3+^-doped (La_0.70_Eu_0.30_FeO_3_) and Eu^3+^/Cr^3+^-co-doped (La_0.70_Eu_0.30_Fe_0.95_Cr_0.05_O_3_) variants are reported.”

In addition, the Acknowledgements section in the original version of this Article was incomplete.

“We thank Sultan Qaboos University (SQU) for providing the PhD scholarship for RTA. We also thank the staff of CAARU, College of Science, SQU, for helping with various measurements. HMW thanks Intisar Sirour for supporting this research in several ways.”

now reads:

“We thank Sultan Qaboos University (SQU) for providing the PhD scholarship for RTA. We also thank the staff of CAARU, College of Science, SQU, for helping with various measurements. HMW thanks Intisar Sirour for supporting this research in several ways. We are thankful to Drs. Ayman Samara and Abdelmajid Salhi of Hamad Bin Khalifa University, Qatar and Prof. Mohamed Henini of the University Nottingham, UK for assistance with magnetic measurements."

The original Article has been corrected.

