# Supplementary material for: Core and surface structure and magnetic properties of mechano-synthesized LaFeO3 nanoparticles and their Eu3+-doped and Eu3+/Cr3+-co-doped variants
Source: Sci Rep. 2024 Jun 26;14:14770. doi: 10.1038/s41598-024-65757-z (PMC11208575; doi:10.1038/s41598-024-65757-z)
Supplement: Supplementary file 1 — Supplementary Figure S1. [file 41598_2024_65757_MOESM1_ESM.docx]

Mechano-synthesized LaFeO_3_ nanoparticles and their Eu^3+^-doped and Eu^3+^/Cr^3+^-co-doped modifications: structural and magnetic studies

R. T. Al‑Mamari^1^, H. M. Widatallah^1.*^, M. E. Elzain^1^, A. M. Gismelseed^1^, A. D. Al‑Rawas^1^,

S. H. Al‑Harthi^1^, M. T. Z. Myint^1^, N. Al-Saqri^1^, M. Al‑Abri^2^

*^1^Physics Department, Sultan Qaboos University, P.O Box 36, Al-Khodh, Muscat 123, Oman*

*^2^ Nanotechnology Research Center, Sultan Qaboos University, P.O Box 17, Oman*

^*^*hishammw@squ.edu.om*

**Supplementary:**


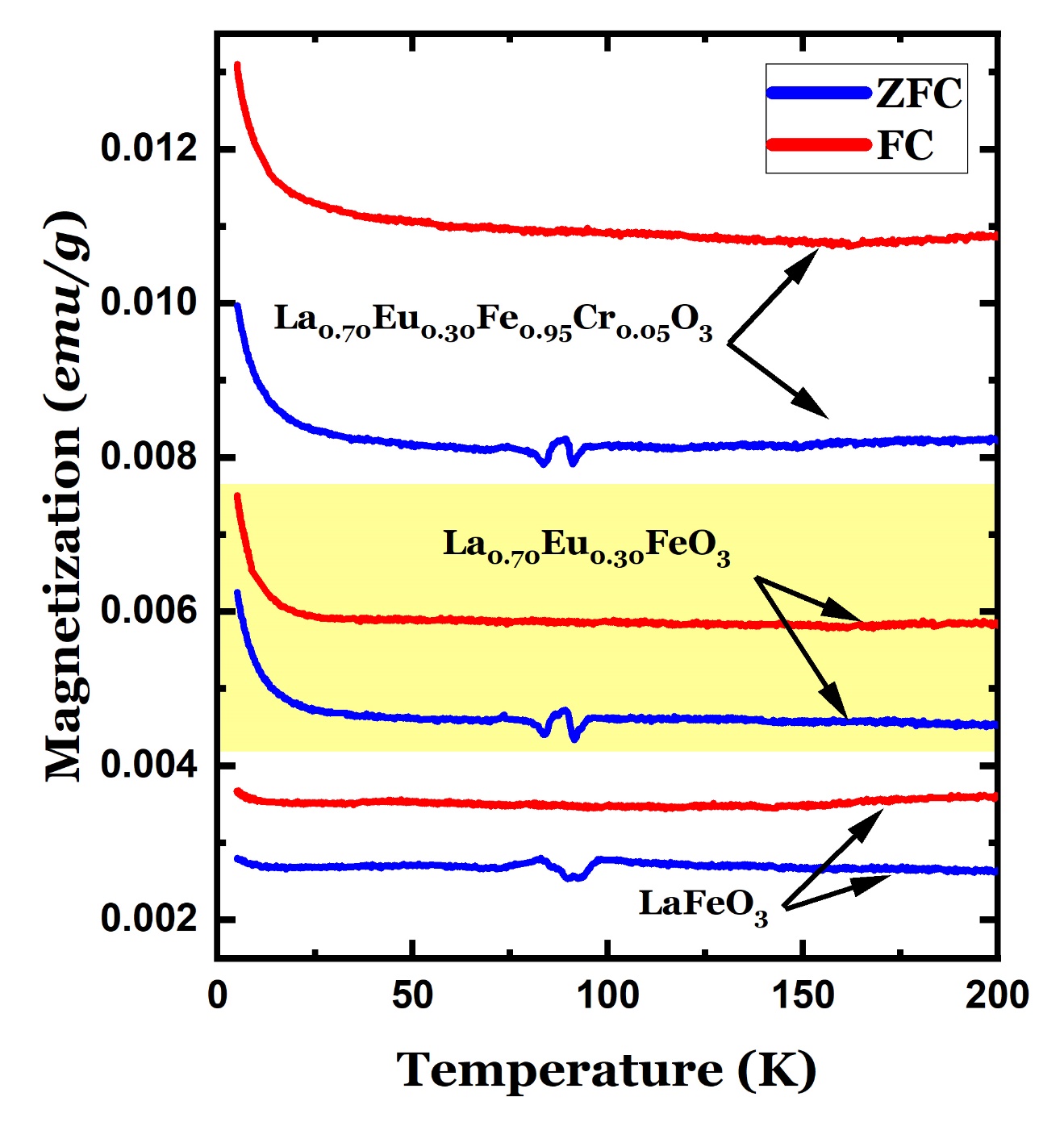


**Figure S1**: The temperature dependence of the FC and ZFC magnetization of the LaFeO_3_, La_0.70_Eu_0.30_FeO_3_, and La_0.70_Eu_0.30_Fe_0.95_Cr_0.05_O_3_ nanoparticles under an applied field of 100 Oe.
